# Assessing runs of Homozygosity: a comparison of SNP Array and whole genome sequence low coverage data

**DOI:** 10.1186/s12864-018-4489-0

**Published:** 2018-01-30

**Authors:** Francisco C. Ceballos, Scott Hazelhurst, Michèle Ramsay

**Affiliations:** 1 Sydney Brenner Institute for Molecular Bioscience, Faculty of Health Sciences, University of the Witwatersrand, Johannesburg, South Africa; 2 Division of Human Genetics, School of Pathology, Faculty of Health Sciences, University of the Witwatersrand, Johannesburg, South Africa; 3 School of Electrical & Information Engineering, University of the Witwatersrand, Johannesburg, South Africa

**Keywords:** Runs of Homozygosity, ROH, SNP array data, WGS low coverage data

## Abstract

**Background:**

Runs of Homozygosity (ROH) are genomic regions where identical haplotypes are inherited from each parent. Since their first detection due to technological advances in the late 1990s, ROHs have been shedding light on human population history and deciphering the genetic basis of monogenic and complex traits and diseases. ROH studies have predominantly exploited SNP array data, but are gradually moving to whole genome sequence (WGS) data as it becomes available. WGS data, covering more genetic variability, can add value to ROH studies, but require additional considerations during analysis.

**Results:**

Using SNP array and low coverage WGS data from 1885 individuals from 20 world populations, our aims were to compare ROH from the two datasets and to establish software conditions to get comparable results, thus providing guidelines for combining disparate datasets in joint ROH analyses. By allowing heterozygous SNPs per window, using the PLINK homozygosity function and non-parametric analysis, we were able to obtain non-significant differences in number ROH, mean ROH size and total sum of ROH between data sets using the different technologies for almost all populations.

**Conclusions:**

By allowing 3 heterozygous SNPs per ROH when dealing with WGS low coverage data, it is possible to establish meaningful comparisons between data using SNP array and WGS low coverage technologies.

**Electronic supplementary material:**

The online version of this article (10.1186/s12864-018-4489-0) contains supplementary material, which is available to authorized users.

## Background

Runs of Homozygosity (ROH) are contiguous regions of the genome where an individual is homozygous across all sites. ROH arise when two copies of an ancestral haplotype are brought together in an individual. Consequently, that haplotype would be autozygous, i.e. homozygous by descent. ROH were first discovered using genome-wide microsatellite scans in the mid 1990s [1]. Members of two families recruited to construct the first human genetic maps carried 4–16 ROH typically 2–40 cM in length; the most extreme individuals had a total of 253 cM in ROH, consistent with close inbreeding. Henceforth, ROH were found to be ubiquitous even in outbred populations; indeed, we are all inbred to some degree and ROH captures this aspect of our individual demographic histories [2–5].

Soon after the first ROH study using short tandem repeat polymorphisms (STRPs) was released, the first SNP arrays started to become available. During those first years, using arrays with densities of 40 K and 120 K SNPs, ROH were discovered to be ubiquitous across all human populations [5]. However, it was not until the first arrays with more than 300 K SNPs were used that the analysis of ROH started to shed light on the understanding of human demographic history and in deciphering the genetic structure of traits and complex diseases [6–8]. Currently array-based genotyping covers around 1.9 to 2.2 million SNPs, allowing meaningful detection of ROH longer than 1 Mb, and even though this is an important improvement over previous arrays, it covers only ~ 2% of the total common SNPs present in the human genome [9, 10]. This prevents the use of array data for detecting shorter ROH, an essential component contributing to the understanding of human genetics. WGS will soon allow shorter ROH to be more reliably called; permitting the effect of very short ROH on diseases risk to be quantified. Thus, analyzing the effect of different lengths of ROH may reveal the relative contributions of multiple rare and common variants to the demographic history of human populations and to explore and test new approaches to understand complex traits [11].

ROH have been a subject of study for understanding human population structures and disease genetics [11–13]. The number and length of ROH reflect individual and population history while the homozygosity burden can be used to investigate the genetic architecture of complex disease. They contributed to studies for different diseases and risk factors, from cancer to cognition, and have been tested for association with either the burden of ROH (total sum of ROH), their abundance (number of ROH), or for association of individual ROH with a phenotype. To date, ROH have been found to be associated with an increased risk of schizophrenia [14, 15], Alzheimer’s disease [16, 17], autism [18, 19], intellectual disabilities [20], lung [21], breast [22] and thyroid cancer [23], and coronary artery disease [24]. In addition, ROH were found to have an effect, in terms of inbreeding depression, on bone mineral density [25], height [12], cognitive ability [26] and education [27]. The application and usefulness of ROH is not limited to humans; ROH have been used to study conservation of endangered species, such as the great apes [28, 29], and to studying inbreeding depression and genomic features in livestock [30, 31]. In view of their usefulness the number of articles published using ROH as a central methodology has recently increased significantly (162 in 2005, 322 in 2010 and 620 in 2016, PubMed search using R package RISmed) and have used predominantly DNA SNP array genotypes. It is expected that, with the current availability of full genome sequences, ROH will be used extensively as an augmentative approach to study population structure, demographic history and in deciphering the genetic structure of complex diseases [13].

The first aim of this article is, therefore, to compare the outcomes and general conclusions drawn for array-based data and low coverage (3-6×) whole genome sequence data from the same groups of individuals. The second is to obtain appropriate parameters of ROH calling that allow meaningful comparison between ROH obtained from both technologies.

There are two major methods for identifying ROH: observational genotype-counting algorithms [32] and model based algorithms [33]. Observational approaches use algorithms that scan each chromosome by moving a fixed size window along the whole length of the genome in search of stretches of consecutive homozygous SNPs [32]. This approach is implemented in PLINK v1.9 where a given SNP is considered to potentially be in an ROH by calculating the proportion of completely homozygous windows that encompass that SNP. If this proportion is higher than a defined threshold, the SNP is designated as being in a ROH. In the algorithm, a variable number of heterozygote positions or missing SNPs can be specified per window in order to tolerate genotyping errors and failures. An ROH is called if the number of consecutive SNPs in a homozygous segment exceeds a predefined threshold in terms of SNP number and/or covered chromosomal length. The simplicity of the approach used by PLINK allows efficient execution on data from large consortia [12]. On the other hand, haplotype-matching algorithms (e.g. Germline) [34] for calculation of identity-by-descent (IBD) can also be used to identify ROH, as a special case of IBD within an individual. Model-based approaches use Hidden Markov Models (HMM) to account for background levels of LD, like the one implemented in Beagle (Browning and Browning [35]). Tests on simulated and real data showed that the approach using PLINK outperformed Germline and Beagle in detecting ROH [36]. This study simulated data by mimicking LD properties in European data, allowing the sequence to resemble expected autozygosity in an outbred European population as well as provide information about true runs of homozygosity. SNP data was obtained from the sequence by sampling common polymorphisms that simulated the allele frequency distribution and SNP density found in modern dense SNP chips.

PLINK, Germline and Beagle software have been used to find ROH in array and WGS data; yet, the HMM model approach is also used with Whole Exome Sequence (WES) data as an alternative to discover SNP variants and small to medium length ROH [37, 38]. However, with the sparse nature of the WES target design, long ROH detection is not possible. Specific software, like “homozygosity heterogeneous hidden Markov model (HMM)” or H^3^M^2^, was designed to deal with this type of data [39].

Accurate ROH calling requires high density SNP genome-wide scan data. A number of factors influence the quality of ROH calling, including the marker density, their distribution across the genome, the quality of the genotype calling/error rates and minor allele frequency. Currently ROH studies have been carried out using genome-wide scan data overwhelmingly from SNP arrays [7, 12, 40], both because of the availability of this data and the fact that array data is considered the gold standard with very low genotyping calling error rates (typically < 0.001). However SNP arrays usually include ~ 1–2.5 million SNP typically with allele frequencies > 0.05, chosen to best represent haplotype structure in target populations. Arrays with more than 300 k SNP genome-wide coverage have been shown to be good enough to successfully detect ROH longer than 1 Mb, which correspond to true ROH arisen by autozygosity [41]. Indeed, it is expected that long ROH will keep their homozygous status independently of the SNP coverage. However, the relative sparsity of SNPs on an array may mean that true heterozygous SNPs between the markers on the array may be missed, thereby making two close-by ROHs appear as one, longer ROH. ROH boundaries will be fuzzier in comparison with WGS and because arrays have fewer SNP they will systematically present and underestimate of ROH shorter than 1 Mb.

A WGS approach, on the other hand, assays every variant so all accessible bases can now be genotyped and more than several million variants, from the most common to the most private can be obtained for each individual [42, 43]. For cost reasons, low coverage sequencing is often employed to maximize the number of participants in a study and strengthen its power. In this case rare SNPs are called significantly less often, with higher error rates, than common SNPs. Whole genome sequence with low-coverage (e.g. 4× average) has a high probability that only one of the two chromosomes of a diploid individual has been sampled at a specific site [42, 43]. Error rates of low coverage WGS can get up to 15% or higher. Of course, reducing and quantifying the uncertainty associated with SNP calling may be accomplished using sophisticated algorithms, and this approach has been subject to extensive research [43]. However, the error rate for low coverage WGS is significantly higher than for array data, which will lead to inaccuracy in ROH calling. This is particularly important, as the cost of WGS becomes more affordable and data more available [44], opening up new possibilities to study ROH in greater detail, replicate results from SNP array data studies, or to the study the relationship of ROH, especially shorter ones, with new populations or traits. Hence, parameters of ROH calling algorithms require tuning to the characteristics of the underlying data in order to obtain meaningful comparable results between studies using different technologies. While in the long run, high coverage data (> 30×) will become more affordable, for the medium-term at least, low-coverage WGS data will be an important source for many analyses.

## Results

### Comparing variant calling between technologies

In order to have a meaningful comparison of ROH obtained from array and WGS low coverage data it is important to first analyze the differences in presence of heterozygous SNPs and variant calling between both technologies. To assess the error rate in heterozygote calling in the WGS, the percentage of concordance in the variant calling between the array and the WGS data, is shown for every population studied (Table 1). As expected, WGS included more heterozygotes SNPs since the SNP array captured only data from ~ 2.5 M nucleotide positions in the autosomal genome, whereas the WGS provided data for the entire length of the genome (~ 2.8 × 10^9^ nucleotide positions). On average, for all the populations analyzed, the WGS low coverage data had 6.3 times more heterozygous SNPs (2,558,000 ± 71,700) compared to the array (404,700 ± 7717) (Table 1). In WGS data there is 1 heterozygous SNP per 1.1 Kb vs 1 in 7.1 Kb in array data. On average the concordance in variant calling by array and WGS is 99.6% (±0.05%). Of the 0.4% (±0.05) discordant calling, on average, 0.1% (±0.03) of the SNPs was called heterozygous by the array and homozygous by WGS and 0.3% (±0.02) of the SNPs was called heterozygous by WGS, but homozygous by array. Considering that array genotyping is the gold standard, WGS data, on average, led to erroneous calling of 0.3% (±0.02) of heterozygous SNP, which would incorrectly be reported as a break in a given ROH. On average, for all the populations, there will be 6500 SNPs (±714) per individual wrongly called as heterozygous, and that is roughly 2.4 SNP (±0.3) per Mb. This error rate is however different across the studied populations, with the JPT having the higher error rate (13,000 wrongly called heterozygotes; 4.5 SNPs per Mb) and the ZUL having the lowest (740 wrongly called heterozygotes; 0.3 SNPs per Mb).

**Table 1:** Mean number of heterozygote SNPs (per called SNP) in array and WGS low coverage data for 20 world populations

			VARIANT CALLING				
	Ave N of Het. WGS	Ave N of Het. Array	Concor.	Discor.	He A − Ho W	Ho A − He W	ROH error
FIN	2,432,921.7	398,280.1	99.6929	0.3071	0.0402	0.2669	0.227
GBR	2,463,526.4	405,223.1	99.6898	0.3102	0.0430	0.2672	0.224
IBS	2,440,125.2	399,870.1	99.6547	0.3453	0.0412	0.3041	0.263
TSI	2,445,524.4	401,124.4	99.6015	0.3985	0.0424	0.3562	0.314
CEU	2,479,523.5	417,837.2	99.6365	0.3635	0.0402	0.3232	0.283
ACB	3,283,726.5	454,173.7	99.6723	0.3277	0.0403	0.2874	0.247
ASW	3,262,716.1	462,107.3	99.6526	0.3474	0.0448	0.3026	0.258
MXL	2,524,698.2	385,362.1	99.7197	0.2803	0.0433	0.2370	0.194
CLM	2,317,649.7	377,844.5	99.6716	0.3284	0.0460	0.2825	0.236
PEL	2,100,245.2	352,485.3	99.6987	0.3013	0.0411	0.2601	0.219
PUR	2,421,174.0	381,603.3	99.4125	0.5875	0.0448	0.5427	0.498
CDX	2,313,375.1	371,361.9	99.7197	0.2803	0.0351	0.2452	0.210
CHB	2,330,226.6	377,553.5	99.7197	0.2803	0.0366	0.2437	0.207
CHS	2,317,649.7	377,844.5	99.6991	0.3009	0.0451	0.2558	0.211
JPT	2,320,417.1	375,586.6	99.3659	0.6341	0.0354	0.5988	0.563
KHV	2,350,584.8	368,521.5	99.8549	0.1451	0.0343	0.1109	0.077
YRI	2,840,113.4	463,890.4	99.5746	0.4254	0.0383	0.3871	0.349
LWK	2,840,253.9	441,435.7	99.5545	0.4455	0.0457	0.3998	0.354
ZUL	2,840,578.1	441,536.6	98.7062	1.2938	0.6338	0.6600	0.026
BAG	2,840,658.6	441,412.3	99.2791	0.7209	0.3157	0.4052	0.090

*Concor.* Concordant**,** *Discor.* Discordant

**He A – Ho W** SNP called heterozygote by array and homozygote by WGS

**Ho A – He W** SNP called homozygote by array and heterozygote by WGS

**ROH error** % of SNPs that being wrongly called can break a ROH

Mean variant calling concordance (in %) is shown. Discordance is discomposed in SNP called heterozygous by array but homozygous by WGS and vice versa. Finally a ROH error is defined as the % of SNP that according to variant calling discordance would break ROH in WGS low coverage data

### Assessing the impact PLINK tolerating heterozygous SNPs in the search for ROH

**Fig. 1:**
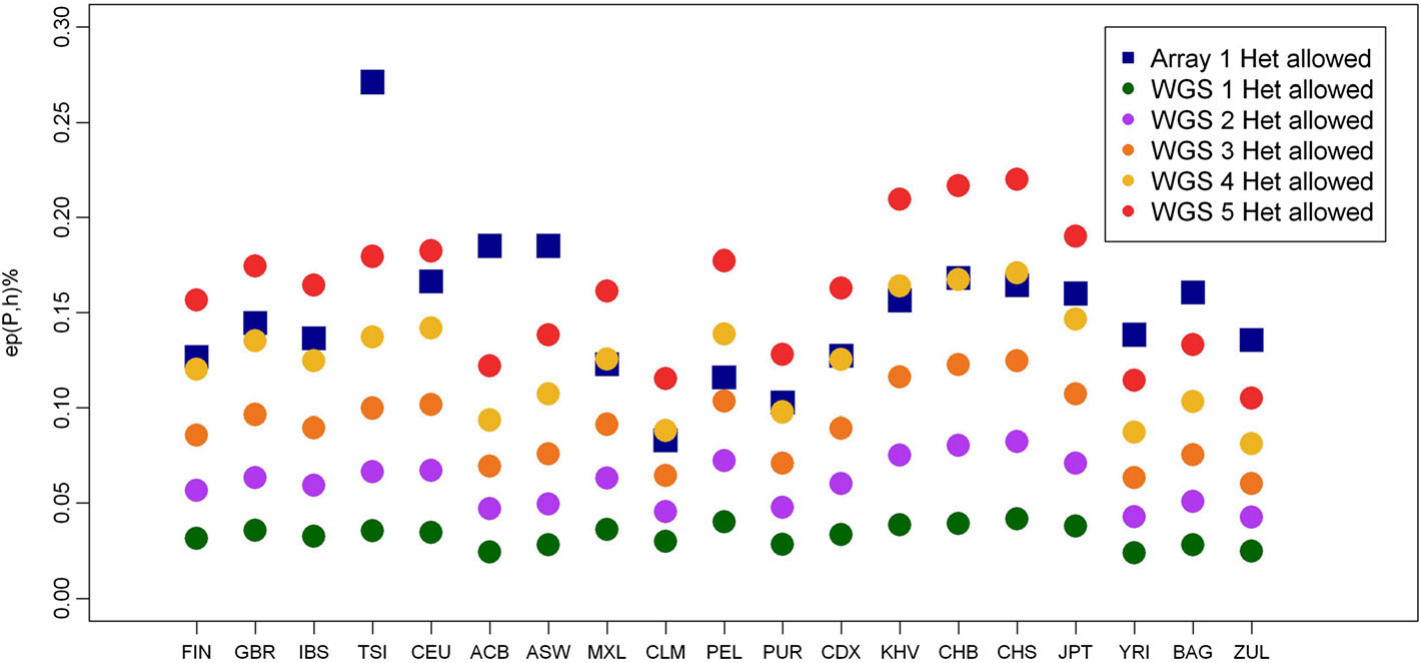
Effect of allowing heterozygous SNPs per window evaluated by *ep(P,h)* as a measure of the empirically observed actually number of heterozygous SNPs found in population *P* when we allow *h* heterozygous SNP. (See Materials and methods for the definition)

Due to its better performance in comparison to other software available, its efficiency execution on data from large consortia and the fact that is the software most used when searching for ROH we use PLINK 1.9 to develop this study. PLINK, by allowing a flexible number of heterozygous SNPs per window (the default value being 1 heterozygous SNP per window), already takes into account possible calling errors that may wrongly break a long ROH. By allowing this heterozygous SNP, the software produces an error that depends on the number of SNP (in homozygous state) per ROH. Figure 1 shows the *ep(P,h)* as a measurement of the empirically observed number of heterozygous SNPs found in ROHs in population *P* when allow *h* heterozygous SNPs per window (1 heterozygous SNP in the array data and 1 to 5 in WGS data, see Materials and methods). This figure shows that for most of the populations the *ep(P,h)* produced by allowing a single heterozygous SNP per window in array data is equivalent to allowing 4 to 5 heterozygous SNPs in WGS data. A few populations deviated from this observation: TSI (0.27% for the array data vs 0.17% after allowing for 5 heterozygous in WGS data), ASW (0.185 vs 0.122), ACB (0.185 vs 0.138), YRI (0.13 vs 0.114), BAG (0.161 vs 0.133) and ZUL (0.136 vs 0.105). These differences are provoked by differences in the mean number of SNPs per ROH as it can be seen in Additional file 1. For example, the TSI population has, on average, 368 SNPs in the homozygous state per ROH in the array data, less than half of the average SNP per ROH in array data across all populations (714.7).

### Obtaining equivalent ROH estimates using data from both technologies

**Fig. 2:**
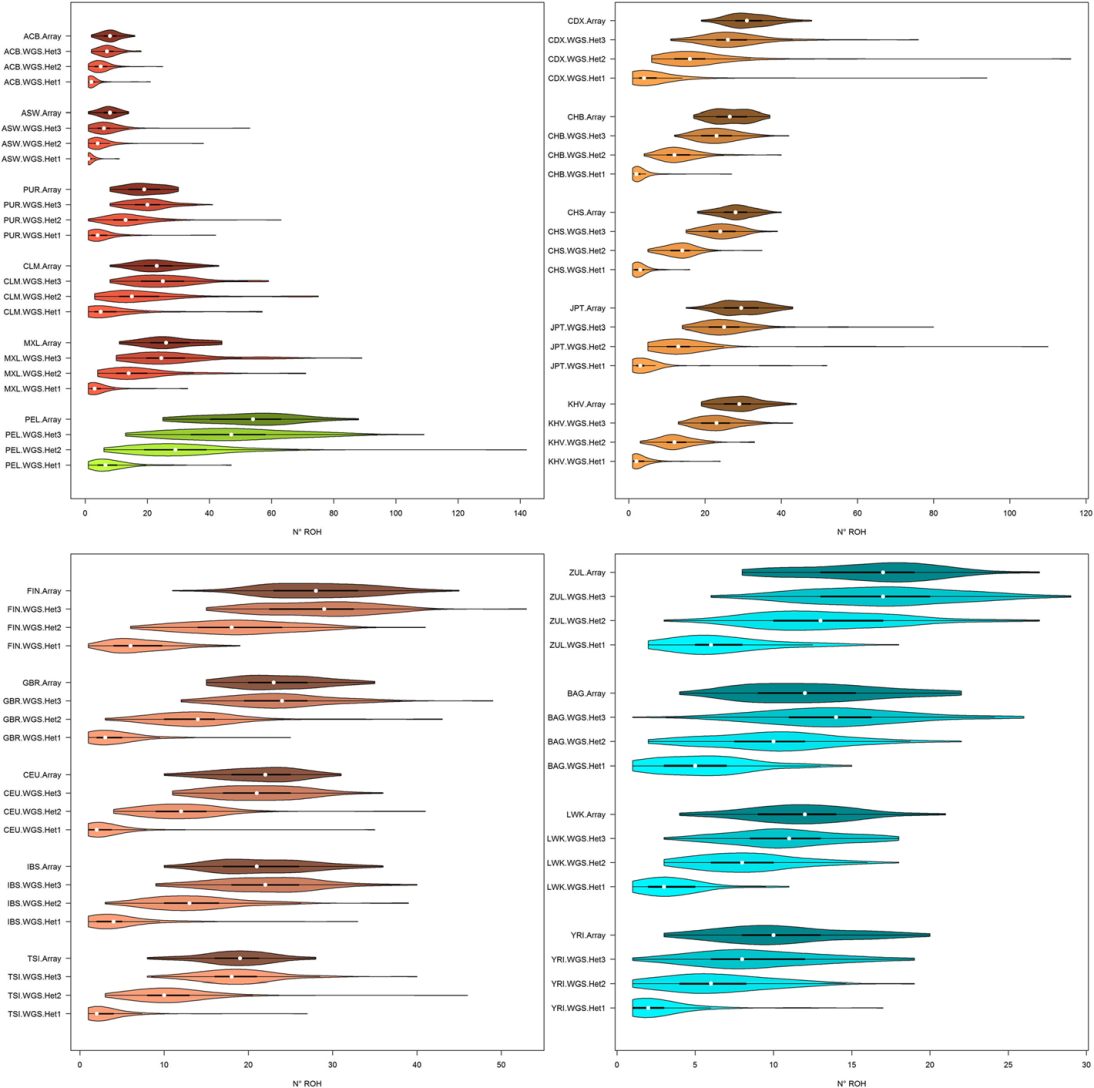
Violin plots of the mean number of ROH longer than 1 Mb. Populations are colored by 5 biogeographical groups by admixture analysis. Admixed (Hispanic-American: CLM, MXL; African-American: ACB, ASW) – blue, Native Americans (PEL) – green, East (CHS, CDX, JPT) and South (KHV) Asia – tan, North (FIN, GBR, CEU) and South (IBS, TSI) Europe – violet, South (ZUL), East (BAG, LWK) and West (YRI) Africa – red. Four distributions per population are shown, array data with 1 heterozygous SNP allowed per window and WGS with 1 to 3 heterozygous SNPs allowed

**Fig. 3:**
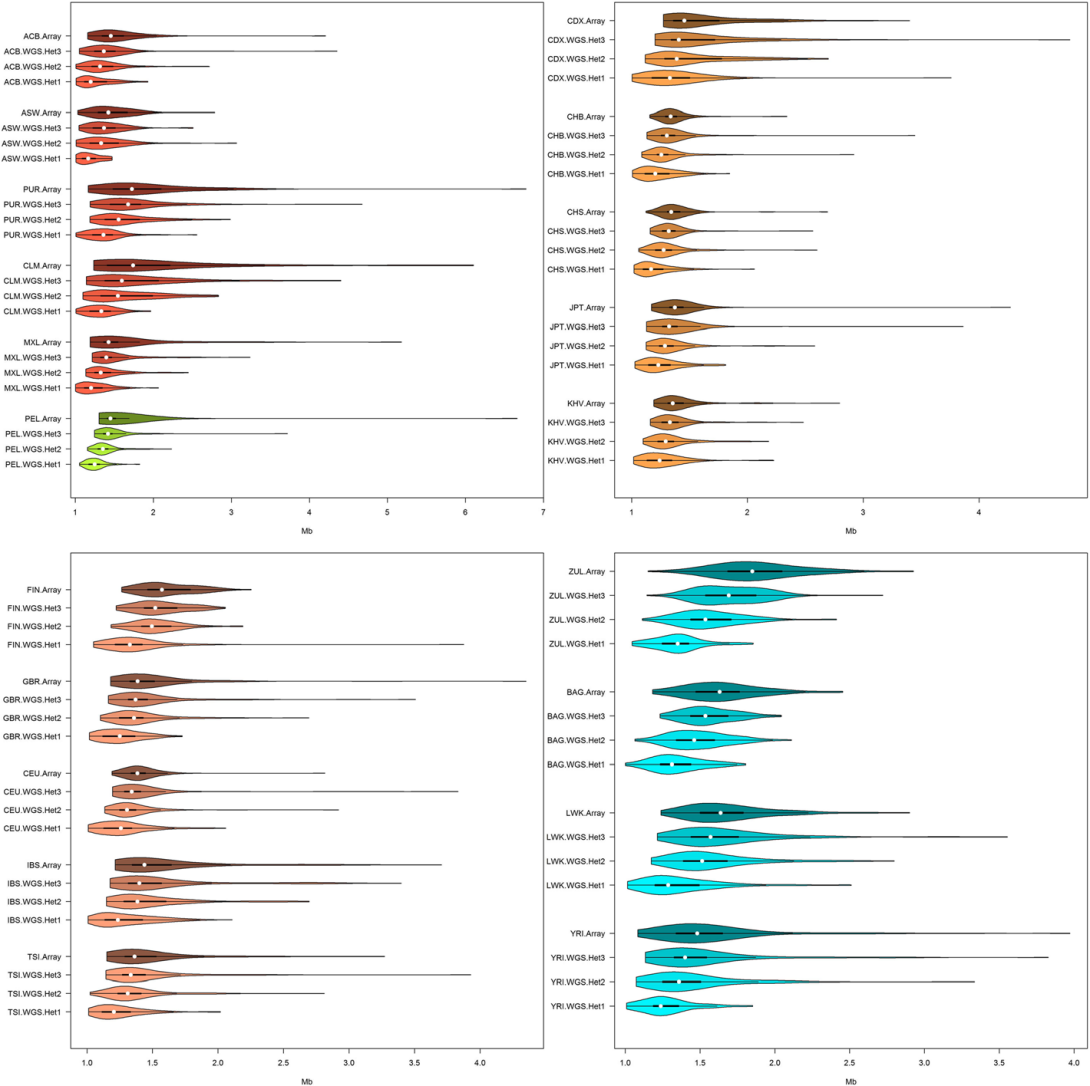
Violin plots of mean ROH size longer than 1 Mb (in Mb). Different biogeographical groups have different *x*-axis scales in an attempt to maximize the difference between distributions within populations. See Fig. 2 legend for population codes

**Fig. 4:**
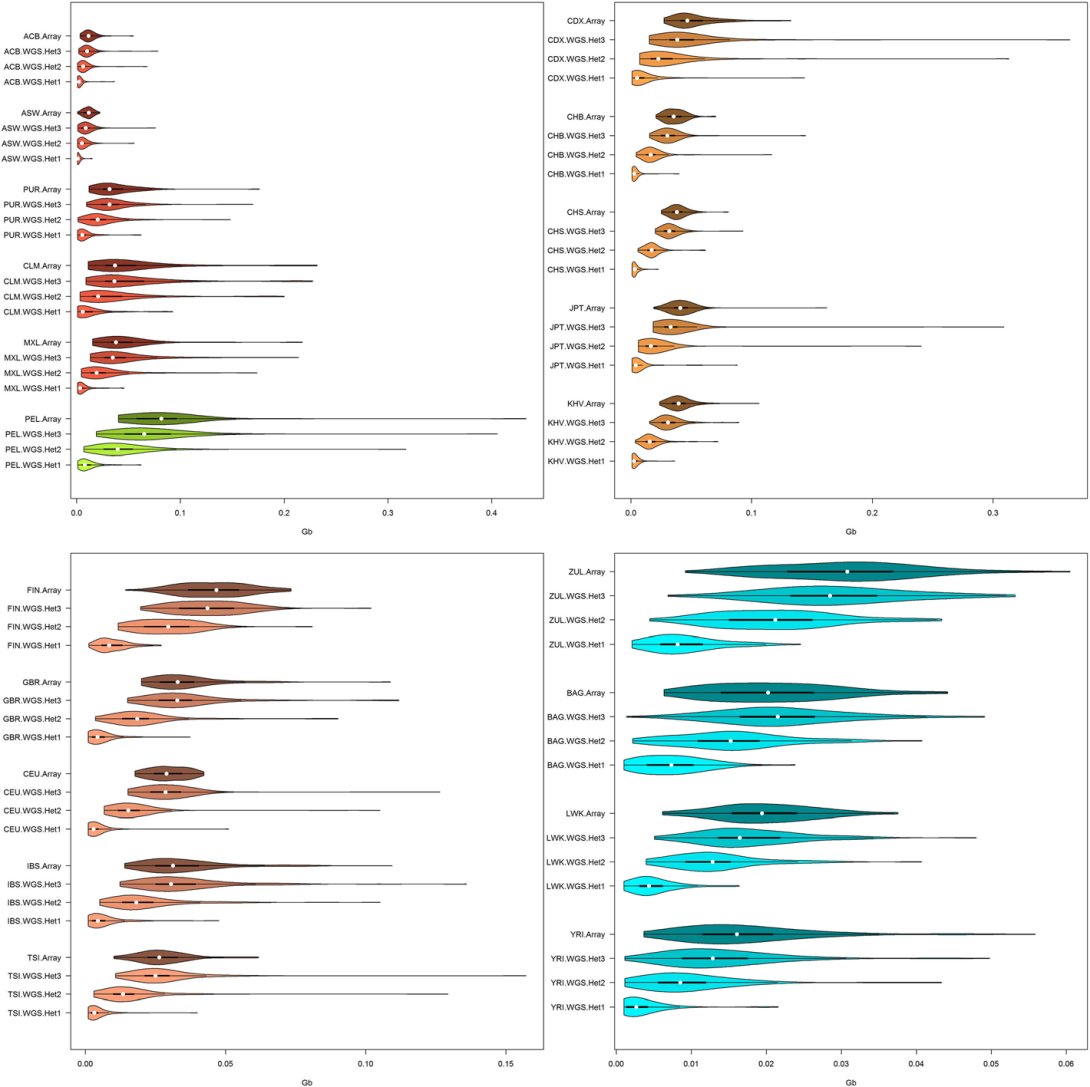
Violin plots of mean total sum of ROH longer than 1 Mb (in Gb). See fig. 2 legend for population codes

**Fig. 5:**
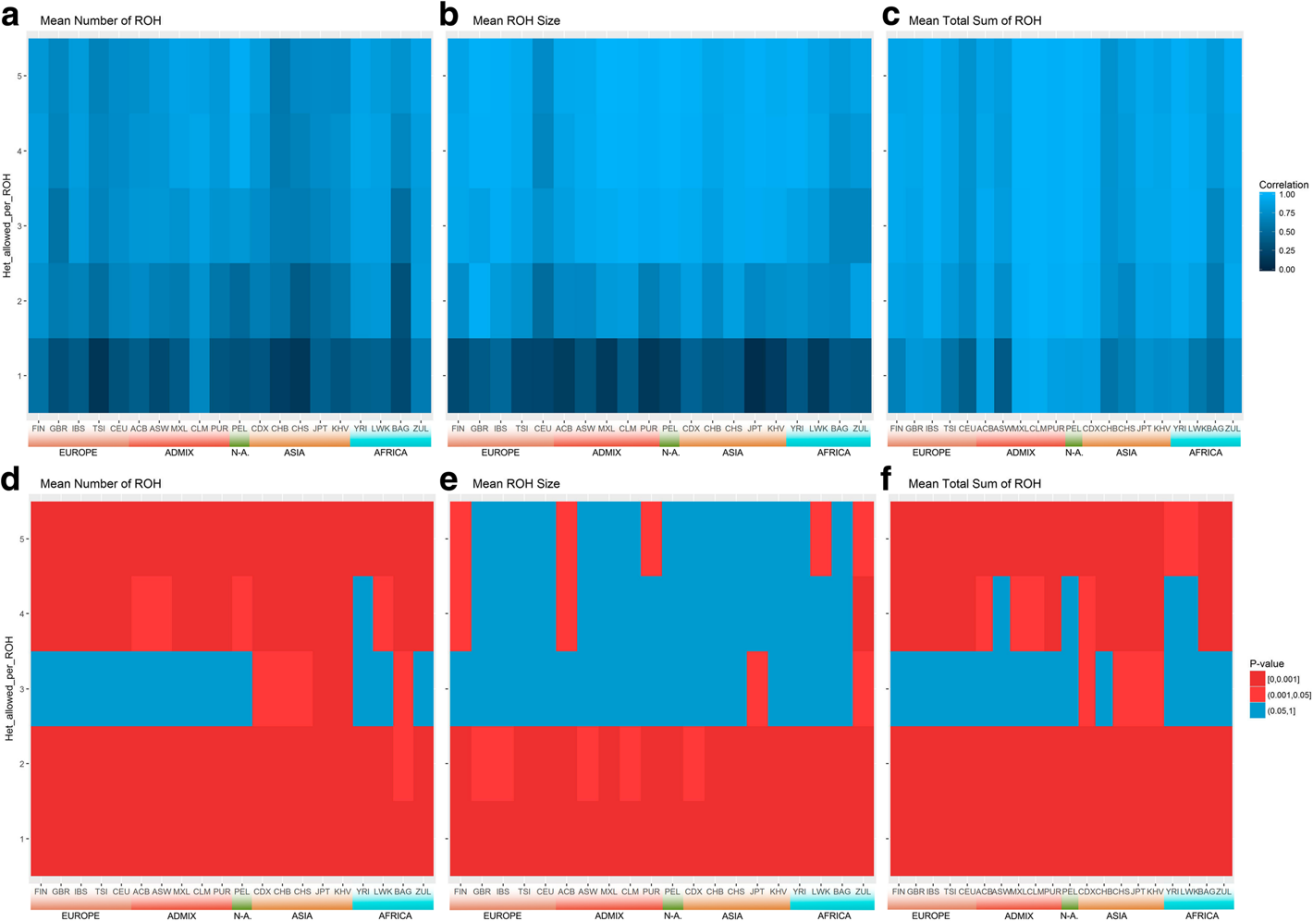
Heatmaps of correlations and MWW tests of mean number of ROH, mean ROH size and mean total sum of ROH between array data allowing 1 heterozygous SNP per window and WGS data allowing 1 to 5 heterozygous SNPs per window (y-axis). **a to c** Pearson correlations. **d to f** *P*-values of Mann-Whitney-Wilcoxon non-parametrical test (MWW), red shows significant difference between array and WGS while blue shows distributions that cannot be considered different. See Fig. 2 legend for population codes

According to both Table 1 and Fig. 1 it seems appropriate to compare ROH from both technologies allowing 1 to 5 heterozygote SNPs in WGS data in order to obtain equivalent results. Violin plots show the distribution of mean number of ROH (Fig. 2), mean ROH size (Fig. 3) and mean total sum of ROH (Fig. 4) per population and using array data, compare to WGS data with 1, 2 or 3 tolerated heterozygotes. Without exception, the distribution between array and WGS data is most similar when 3 heterozygous SNPs in the WGS data are allowed per window. Mean values and standard deviations for up to 5 heterozygous SNPs allowed per window are shown in Additional file 2. Figure 5a–c show the correlations with the array data as heat-maps between number of ROH (5a), mean ROH size (5b), and total sum of ROH (5c) for each population and a different number of allowed heterozygous SNPs in the WGS data (values and probabilities shown in Additional file 3). The correlations, as expected, increase with more heterozygous SNPs being allowed in the WGS data. Correlations are not homogeneous, south and East Asian populations show lower correlations in comparison with other populations. An alternative representation by line charts is shown in Additional file 4, where differences between populations are perceived more easily. In addition, non-parametrical statistical analysis was used. Results of the statistical comparison between ROH obtained from array and WGS (with a different number of heterozygous SNPs allowed) by the Mann-Whitney-Wilcoxon (MWW) test are shown as a heat-map of significance (*p* values; blue = not significant) in Figs. 5d–f. *P*-values are presented in Additional file 3. These figures show heterogeneous results across populations. In general, by allowing 3 heterozygotes SNPs per window in WGS the statistical outcomes in the number of ROH, mean ROH size and total sun of ROH are similar between array and WGS data. However, Fig. 5d–f also show that for the Asian populations, especially the JPT, for the number of ROH and total sum of ROH differences between array and WGS data are significant for every heterozygous SNP allowed.

### Comparing ROH with different lengths

**Fig. 6:**
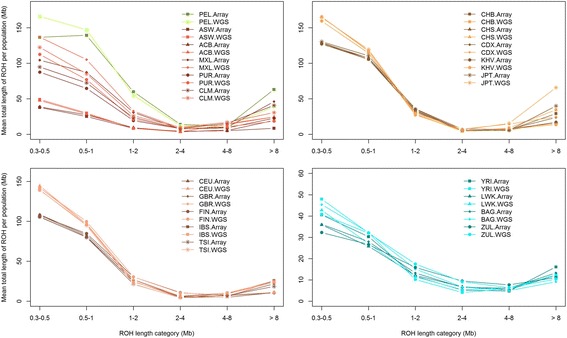
Mean sum of ROH in different length categories. The light colored lines represent WGS with 3 heterozygous SNP allowed per window and dark colored lines represent array data with 1 heterozygous SNP allowed per ROH. See Fig. 2 legend for population codes

Once we established that the best PLINK condition to obtain comparable results is to allow 3 heterozygous SNPs per window when dealing with WGS low coverage, we compared the mean sum of ROH in both technologies for different ROH length categories (Fig. 6). This is relevant because the study of different ROH lengths has different applications, as indicated in Table 2. Figure 6 shows that for ROH longer than 1 Mb, the array and WGS mean total lengths are very similar, with some exceptions like the JPT, in the case of ROH longer than 8 Mb. However, WGS data systematically detected more short ROH (0.3 – 1 Mb) than array data. This outcome is expected and is caused by the lower SNP coverage of array data, since PLINK considered just ROH containing at least 50 SNPs. This gap between array and WGS data can be corrected for small ROH by changing PLINK parameters and relaxing the number of SNPs needed to call a ROH (−-homozyg-snp 30, data not shown).

**Table 2:** Performance of different technologies (array, WGS low coverage and WES) with different ROH size classes (Short < 1 Mb, Medium 1 – 8 Mb and Long > 8 Mb)

ROH size Class	SNP Array	WGS low coverage	WES	Applications
Short < 1 Mb	Poor performance due to low SNP coverage. Can be adjusted to detect ROH by modifying the number of SNPs required in a ROH.	Able to detect but need to build adjustment for genotype calling errors.	Able to detect but only in selected genomic regions. Software like H^3^M^2^ allows meaningful regional analysis [39].	Detection of rare variants involved in deleterious recessive alleles and directional dominance [11, 12]. Analysis of LD patterns and extreme bottle necks [33].
Medium 1-8 Mb	Able to detect if the array has at least 300 K SNPs. ROH boundaries will be fuzzier in comparison with WGS low coverage data.	Good performance but need to build adjustment for genotype calling errors. Allowing 3 heterozygous SNPs per ROH would grant meaningful outcomes.	Able to detect, but only in selected genomic regions and boundaries of ROH could be fuzzy if they reach into non-exonic regions [49].	Detection of rare variants involved in diseases. Analysis of inbreeding depression. Genome architecture and ROH island detection [50]. Population history, bottle necks, remote consanguinity and genetic drift [51].
Long > 8 Mb	Good performance if the array has at least 300 K SNPs.	Good performance but need to build adjustment for genotype calling errors. Allowing 3 heterozygous SNPs per ROH would grant meaningful outcomes.	Poor performance due to short size of most exons and their sparsity across the genome.	Analysis of inbreeding depression. Validation of GWAS findings. Population history and cultural practices, close consanguinity [6, 41].

## Discussion

Runs of homozygosity are an excellent tool to delve into the exploration of different aspects of human genetics. Large genomic datasets, using array and whole genome sequence data, are now becoming available and offer the researcher a unique opportunity to better understand the influence of ROH on complex diseases architecture and demographic history.

Ideally, WGS deep coverage would be the best option to study ROH, since genotype calling will be robust for low MAFs and ROH of virtually any size would be detected. However, two major issues prevent the use this technology. First, the lack of WGS deep coverage data for population studies and secondly, the extreme computational expense of analyzing this type of data using current software. Unlike deep coverage, low coverage WGS data is more abundant and affordable, and the computational effort of obtaining ROH is less computationally intensive. The only drawback of using this data is the calling error associated with it. By comparing ROH obtained from array data, we demonstrate in this article that this problem can be mitigated by allowing 3 heterozygous SNPs per window using PLINK software to obtain ROH longer than 1 Mb. In all populations, the highest correlation was achieved when allowing 3 to 4 heterozygous SNPs per window (Fig. 5a–c). Regarding MWW tests (Fig. 5d–f), unlike mean number and total sum of ROH, for most of the populations, mean ROH size remains equivalent between technologies when allowing 3 or more heterozygous SNPs per window. As expected, we get more ROH by allowing more heterozygous SNPs, but the mean size remains constant. As a consequence, the mean total sum of ROH increases with more heterozygous SNP allowed.

Interestingly, four populations from East and South Asia did not conform to the patterns observed in the other populations; in fact for the Dai and Han populations from China (CDX, CHS), Kinh population from Vietnam (KHV) and the Japanese population (JPT), it was not possible to obtain the same mean number and total sum of ROH between array and WGS data. This may be explained by population structure, but perhaps the inferior performance of the Infinium Omni 2.5–8 Bead chip in Asian populations [45] is the more plausible explanation. This could also explain why it was not possible to obtain same number of ROH in the Baganda population from Uganda (BAG) or the same mean ROH size in the Zulu population from South Africa (ZUL).

WGS data present the ability to identify shorter ROH (Fig. 6), however it would be important to compare the short ROH detected using low coverage, compared to high coverage data to establish a comparative analysis guideline. In Table 2 we present a comparison in performance of the application of three different technologies (SNP array, WGS low coverage and WES data) to detect short, medium and long ROH.

## Conclusions

This study provides evidence-based guidelines for the combined analysis of array and low coverage WGS data when studying ROH to investigate population history and to detect associations with complex diseases and traits.

We demonstrate that, even though there are differences between populations around the world, is possible to get equivalent results between WGS low coverage and SNP array technologies by allowing 3 heterozygous SNP per window when dealing with WGS low coverage data.

## Methods

### Description of data

Individuals with both genome-wide SNP genotypic data and WGS low coverage data from the 1000 Genomes Project – Phase 3 (KGP) [46, 47] and the African Genome Variation Project (AGVP) [48] were used. For both datasets the Infinium Omni 2.5–8 Bead chip from Illumina was used. The KGP includes a total of 1685 individuals from 18 populations with genotypic data available from array and WGS low coverage (4×). From Europe: FIN (Finish in Finland, *n* = 99), GBR (British in England and Scotland, *n* = 91), IBS (Iberian populations in Spain, *n* = 105), TSI (Tuscany in Italy, *n* = 102) and CEU (Utah residents with European ancestry = 99). From America: ASW (Americans of African ancestry in Huston, *n* = 61), ACB (African Caribbean in Barbados, *n* = 96), PUR (Puerto Rican in Puerto Rico with admix ancestry, *n* = 104), PEL (Peruvian in Lima, Peru with Amerindian ancestry, *n* = 85), CLM (Colombian in Medellin, Colombia with admix ancestry, *n* = 95) and MXL (Mexican with admix ancestry in Los Angles, USA, *n* = 100). From Asia: CDX (Chinese Han in Xishuangbanna, China, *n* = 98), CHB (Chinese Han in Beijing, China, n = 100), CHS (Southern Han Chinese, n = 105), JPT (Japanese in Tokio, Japan, n = 100) and KHV (Kinh in Ho Chi Minh city, Vietnam n = 99). From Africa: YRI (Yoruba in Ibadan, Nigeria, *n* = 108) and LWK (Luhya in Webuye, Kenya, n = 99). The AVGP includes 2185 samples from 16 African populations; we use WGS data for two: 100 Zulu from South Africa and 100 Baganda from Uganda, where genotype data from the Omni 2.5–8 SNP array and WGS data at 4× coverage are available. Only SNPs of the 22 autosomes were included in this analysis. For each population, data from both array genotyping and WGS were filtered to remove SNP with minor allele frequencies lower than 0.05 and those that divert from H-W proportions with *p* < 0.001. This filtering limits the effects of ascertainment bias caused by the small number of individuals in the SNP discovery panel, in the case of the array, and the calling errors associated with a low depth coverage of whole genome sequence data.

Identification and Characterization of ROH:

We used PLINK v1.9 to identify ROH. The following conditions were used:

- *homozyg-snp 50.* Minimum number of SNPs that a ROH is required to have
- *homozyg-kb 300.* Length in Kb of the sliding window
- *hmozyg-density 50.* Required minimum density to consider a ROH (1 SNP in 50Kb)
- *homozyg-gap 1000.* Length in Kb between two SNPs in order to be considered in two different segments.
- *homozyg-window-snp 50.* Number of SNPs that the sliding window must have
- *homozyg-window-het (1 to 5).* Number of heterozygous SNP allowed in a window
- *homozyg-window-missing 5.* Number of missing calls allowed in a window
- *homozyg-window-threshold 0.05.* Proportion of overlapping windows that must be called homozygous to define a given SNP as in a “homozygous” segment.

The minimum length of a ROH was set to 300 kb. PLINK allows the setting of different variable number of heterozygous SNPs per window, with a default value of 1 heterozygous genotype per window, in order to tolerate genotyping calling errors (*−-homozyg-window-het 1*). This is especially relevant in dealing with WGS low coverage data and therefore we were testing the equivalence between ROH obtained from array genotyping and WGS data.

### Assessing the impact tolerating heterozygous SNPs while using PLINK in the search for ROH

Our goal is to determine under which conditions detecting ROHs using low coverage sequence data results in comparable results as using SNP array data. There are several characteristics of ROHs we can measure. To be in a position to combine datasets generated by different technologies, we need to identify characteristics to allow their joint assessment, no matter the technology used.

The effect of allowing different numbers of heterozygous SNPs per ROH can be evaluated in different ways. We define *ep(P,h)* as a measure of the empirically observed actual number of heterozygous SNPs found in population *P* when we allow *h* heterozygous SNPs.

- Let *R(P,h,x,y)* be the set of ROHs with length in the range *[x,y)* in population *P* when allowing *h* heterozygous SNP.
- Let |*R(P,h,x,y)*| be the number of ROHs of length in the range *[x,y)* in population *P* when allowing *h* heterozygote
- Let *ep*_*xy*_*(P,h)* = arithmetic mean of the actual number of heterozygous found in all ROHs in *R(P,h,x,y)* found in the population under study
- Finally,
  $$ ep\left(P,h\right)=\frac{\sum_{xy}\left|R\Big(P,h,x,y\right|{ep}_{xy}\left(P,h\right)}{\sum_{xy}\left|R\Big(P,h,x,y\right|}\ x\ 100 $$

We sum over (x,y) є {[1,1.5),[1.5,5),[5,10),[10,∞)}.

This observed number of heterozygous SNPs differs from the parameter used for detecting ROHs depending on the population and technology platform characteristics.

### Statistical analysis

For comparison purposes three variables per population were defined. Mean number of ROH as the mean number of ROH longer than 1 Mb. Mean ROH size as the mean size of ROH longer than 1 Mb. Total sum of ROH as the mean total sum of ROH longer than 1 Mb. Considering just ROH longer than 1 Mb allows the selection of only the ROH arising from identity by descent and to remove any LD effects. Data distributions were illustrated using violin plots. This plot combines a box plot with a kernel density plot, where the interval width is obtained by the rule of thumb. The violin shows a colored kernel density trace with the interquartile range as a black line and median as a white dot. This representation is especially relevant when dealing with data or variables that show skewed distributions and is a good means of comparison between populations, when dealing with asymmetric distributions where the median is more informative than the mean. Statistical comparisons between mean number of ROH, mean ROH size and mean total sum of ROH for different populations, technologies and PLINK conditions were performed by Pearson’s correlation and Mann-Whitney-Wilcoxon non-parametric test (MWW). All the exploratory and statistical analyses were performed using R.

## Additional files

Additional file 1: Table S1. Mean number of SNP (in homozygous state) per ROH in array data with 1 heterozygous SNP per ROH and WGS data with 1 to 5 heterozygous SNPs per ROH. *ep(P,h)* values for different populations *P* and allowed heterozygous SNP. (DOCX 22 kb)

Additional file 2: Table S2. Pearson correlations (y-axis) of number of ROH, mean ROH size and mean sum of ROH between array data with 1 heterozygous allowed per RHO and WGS with 1 to 5 heterozygous SNP allowed per ROH (x-axis). (DOCX 28 kb)

Additional file 3: Table S3. Means and standard deviations of number of ROH, ROH size and total sum of ROH for different populations, technologies and allowed heterozygous SNPs per ROH (DOCX 35 kb)

Additional file 4: Fig. S1. Pearson correlations (with *p*-values) and Mann-Whitney-Wilcoxon non- parametrical test p vales between array data with 1 heterozygous SNP per ROH and WGS with 1 to 5 heterozygous SNPs per ROH. (JPEG 1011 kb)

## References

[1] Broman KW, Weber JL (1999). Long homozygous chromosomal segments in reference families from the centre d'Etude du polymorphisme humain. Am J Hum Genet.

[2] Keller MC, Visscher PM, Goddard ME (2011). Quantification of inbreeding due to distant ancestors and its detection using dense single nucleotide polymorphism data. Genetics.

[3] Biraben J-N (1980). An essay concerning mankind's demographic evolution. J Hum Evol.

[4] Donnelly KP (1983). The probability that related individuals share some section of genome identical by descent. Theor Popul Biol.

[5] Gibson J, Morton NE, Collins A (2006). Extended tracts of homozygosity in outbred human populations. Hum Mol Genet.

[6] McQuillan R, Eklund N, Pirastu N, Kuningas M, McEvoy BP, Esko T, Corre T, Davies G, Kaakinen M, Lyytikainen LP (2012). Evidence of inbreeding depression on human height. PLoS Genet.

[7] Ku CS, Naidoo N, Teo SM, Pawitan Y, Kuningas M, McQuillan R, Wilson JF, Hofman A, van Duijn CM, Uitterlinden AG (2011). Regions of homozygosity and their impact on complex diseases and traits runs of homozygosity do not influence survival to old age. Nat Rev.

[8] Wang JC, Ross L, Mahon LW, Owen R, Hemmat M, Wang BT, El Naggar M, Kopita KA, Randolph LM, Chase JM (2015). Regions of homozygosity identified by oligonucleotide SNP arrays: evaluating the incidence and clinical utility. Eur J Hum Genet.

[9] LaFramboise T (2009). Single nucleotide polymorphism arrays: a decade of biological, computational and technological advances. Nucleic Acids Res.

[10] Lamy P, Grove J, Wiuf C (2011). A review of software for microarray genotyping. Hum Genomics.

[11] Szpiech ZA, Xu J, Pemberton TJ, Peng W, Zollner S, Rosenberg NA, Li JZ (2013). Long runs of homozygosity are enriched for deleterious variation. Am J Hum Genet.

[12] Joshi PK, Esko T, Mattsson H, Eklund N, Gandin I, Nutile T, Jackson AU, Schurmann C, Smith AV, Zhang W (2015). Directional dominance on stature and cognition in diverse human populations. Nature.

[13] Ceballos F.C., Joshi P.K., Clark D.W., Ramsay M. and Wilson J.F. Runs of homozygosity: windows into population history and trait architecture. Nat. Rev. Genet. 2018. https://dx.doi.org/10.1038/nrg.2017.109. 10.1038/nrg.2017.109 29335644

[14] Lencz T, Lambert C, DeRosse P, Burdick KE, Morgan TV, Kane JM, Kucherlapati R, Malhotra AK (2007). Runs of homozygosity reveal highly penetrant recessive loci in schizophrenia. Proc Natl Acad Sci U S A.

[15] Keller MC, Simonson MA, Ripke S, Neale BM, Gejman PV, Howrigan DP, Lee SH, Lencz T, Levinson DF, Sullivan PF (2012). Runs of homozygosity implicate autozygosity as a schizophrenia risk factor. PLoS Genet.

[16] Nalls MA, Guerreiro RJ, Simon-Sanchez J, Bras JT, Traynor BJ, Gibbs JR, Launer L, Hardy J, Singleton AB (2009). Extended tracts of homozygosity identify novel candidate genes associated with late-onset Alzheimer's disease. Neurogenetics.

[17] Ghani M, Reitz C, Cheng R, Vardarajan BN, Jun G, Sato C, Naj A, Rajbhandary R, Wang LS, Valladares O (2015). Association of Long Runs of Homozygosity with Alzheimer disease among African American individuals. JAMA Neurol.

[18] Chahrour MH, Yu TW, Lim ET, Ataman B, Coulter ME, Hill RS, Stevens CR, Schubert CR, Greenberg ME, Gabriel SB (2012). Whole-exome sequencing and Homozygosity analysis implicate depolarization-regulated neuronal genes in autism. PLoS Genet.

[19] Lin PI, Kuo PH, Chen CH, Wu JY, Gau SS, Wu YY, Liu SK: Runs of homozygosity associated with speech delay in autism in a taiwanese han population: evidence for the recessive model. PLoS One 2013, 8(8):e72056. 10.1371/journal.pone.0072056 PMC3745408 23977206

[20] Gamsiz ED, Viscidi EW, Frederick AM, Nagpal S, Sanders SJ, Murtha MT, Schmidt M, Triche EW, Geschwind DH, State MW (2013). Intellectual disability is associated with increased runs of homozygosity in simplex autism. Am J Hum Genet.

[21] Orloff MS, Zhang L, Bebek G, Eng C (2012). Integrative genomic analysis reveals extended germline homozygosity with lung cancer risk in the PLCO cohort. PLoS One.

[22] Thomsen H, Filho MI, Woltmann A, Johansson R, Eyfjord JE, Hamann U, Manjer J, Enquist-Olsson K, Henriksson R, Herms S (2015). Inbreeding and homozygosity in breast cancer survival. Sci Rep.

[23] Thomsen H, Chen B, Figlioli G, Elisei R, Romei C, Cipollini M, Cristaudo A, Bambi F, Hoffmann P, Herms S (2016). Runs of homozygosity and inbreeding in thyroid cancer. BMC Cancer.

[24] Christofidou P, Nelson CP, Nikpay M, Qu L, Li M, Loley C, Debiec R, Braund PS, Denniff M, Charchar FJ (2015). Runs of Homozygosity: association with coronary artery disease and gene expression in Monocytes and macrophages. Am J Hum Genet.

[25] Yang TL, Guo Y, Zhang JG, Xu C, Tian Q, Deng HW (2015). Genome-wide survey of runs of Homozygosity identifies recessive loci for bone mineral density in Caucasian and Chinese populations. J Bone Miner Res.

[26] Howrigan DP, Simonson MA, Davies G, Harris SE, Tenesa A, Starr JM, Liewald DC, Deary IJ, McRae A, Wright MJ (2016). Genome-wide autozygosity is associated with lower general cognitive ability. Mol Psychiatry.

[27] Abdellaoui A, Hottenga JJ, Willemsen G, Bartels M, van Beijsterveldt T, Ehli EA, Davies GE, Brooks A, Sullivan PF, Penninx BW (2015). Educational attainment influences levels of homozygosity through migration and assortative mating. PLoS One.

[28] Xue Y, Prado-Martinez J, Sudmant PH, Narasimhan V, Ayub Q, Szpak M, Frandsen P, Chen Y, Yngvadottir B, Cooper DN (2015). Mountain gorilla genomes reveal the impact of long-term population decline and inbreeding. Science.

[29] Prado-Martinez J, Sudmant PH, Kidd JM, Li H, Kelley JL, Lorente-Galdos B, Veeramah KR, Woerner AE, O'Connor TD, Santpere G (2013). Great ape genetic diversity and population history. Nature.

[30] Chitneedi PK, Arranz JJ, Suarez-Vega A, Garcia-Gamez E, Gutierrez-Gil B (2017). Estimations of linkage disequilibrium, effective population size and ROH-based inbreeding coefficients in Spanish Churra sheep using imputed high-density SNP genotypes. Anim Genet.

[31] Purfield DC, McParland S, Wall E, Berry DP (2017). The distribution of runs of homozygosity and selection signatures in six commercial meat sheep breeds. PLoS One.

[32] Purcell S, Neale B, Todd-Brown K, Thomas L, Ferreira MAR, Bender D, Maller J, Sklar P, de Bakker PIW, Daly MJ (2007). PLINK: a tool set for whole-genome association and population-based linkage analyses. Am J Hum Genet.

[33] Pemberton TJ, Absher D, Feldman MW, Myers RM, Rosenberg NA, Li JZ (2012). Genomic patterns of homozygosity in worldwide human populations. Am J Hum Genet.

[34] Gusev A, Lowe JK, Stoffel M, Daly MJ, Altshuler D, Breslow JL, Friedman JM, Pe'er I (2009). Whole population, genome-wide mapping of hidden relatedness. Genomic Res.

[35] Browning SR, Browning BL (2010). High-resolution detection of identity by descent in unrelated individuals. Am Jour Hum Genetics..

[36] Howrigan DP, Simonson MA, Keller MC (2011). Detecting autozygosity through runs of homozygosity: a comparison of three autozygosity detection algorithms. BMC Genomics.

[37] Zhuang Z, Gusev A, Cho J, Pe'er I (2012). Detecting identity by descent and homozygosity mapping in whole-exome sequencing data. PLoS One.

[38] Mezzavilla M, Vozzi D, Badii R, Alkowari MK, Abdulhadi K, Girotto G, Gasparini P, McLaughlin RL, Kenna KP, Vajda A (2015). Increased rate of deleterious variants in long runs of homozygosity of an inbred population from Qatar. Hum Hered.

[39] Magi A, Tattini L, Palombo F, Benelli M, Gialluisi A, Giusti B, Abbate R, Seri M, Gensini GF, Romeo G (2014). H3M2: detection of runs of homozygosity from whole-exome sequencing data excess of homozygosity in the major histocompatibility complex in schizophrenia. Bioinformatics.

[40] Yang HC, Chang LC, Liang YJ, Lin CH, Wang PL (2012). A genome-wide homozygosity association study identifies runs of homozygosity associated with rheumatoid arthritis in the human major histocompatibility complex. PLoS One.

[41] McQuillan R, Leutenegger AL, Abdel-Rahman R, Franklin CS, Pericic M, Barac-Lauc L, Smolej-Narancic N, Janicijevic B, Polasek O, Tenesa A (2008). Runs of homozygosity in European populations. Am J Hum Genet.

[42] Nielsen R, Paul JS, Albrechtsen A, Song YS (2011). Genotype and SNP calling from next-generation sequencing data. Nat Rev Genet.

[43] Goodwin S, McPherson JD, McCombie WR, Purcell S, Neale B, Todd-Brown K, Thomas L, Ferreira MA, Bender D, Maller J (2016). Coming of age: ten years of next-generation sequencing technologies PLINK: a tool set for whole-genome association and population-based linkage analyses. Nat Rev Genet.

[44] DNA sequencing cost: data from the NHGRI Genome Sequencing Program (GSP) [http://www.genome.gov/sequencingcosts].

[45] Ha NT, Freytag S, Bickeboeller H (2014). Coverage and efficiency in current SNP chips. Eur J Hum Genet.

[46] The 1000 Genomes Project C (2015). A global reference for human genetic variation. Nature.

[47] Sudmant PH, Rausch T, Gardner EJ, Handsaker RE, Abyzov A, Huddleston J, Zhang Y, Ye K, Jun G, Hsi-Yang Fritz M (2015). An integrated map of structural variation in 2,504 human genomes. Nature.

[48] Gurdasani D, Carstensen T, Tekola-Ayele F, Pagani L, Tachmazidou I, Hatzikotoulas K, Karthikeyan S, Iles L, Pollard MO, Choudhury A (2015). The African genome variation project shapes medical genetics in Africa. Nature.

[49] Pippucci T, Magi A, Gialluisi A, Romeo G, Hosking FJ, Papaemmanuil E, Sheridan E, Kinsey SE, Lightfoot T, Roman E (2014). Detection of runs of homozygosity from whole exome sequencing data: state of the art and perspectives for clinical, population and epidemiological studies genome-wide homozygosity signatures and childhood acute lymphoblastic leukemia risk single nucleotide polymorphism arrays: a decade of biological, computational and technological advances. Hum Hered.

[50] Curtis D, Vine AE, Knight J (2008). Study of regions of extended homozygosity provides a powerful method to explore haplotype structure of human populations. Ann Hum Genet.

[51] Nothnagel M, Lu TT, Kayser M, Krawczak M, Spain SL, Cazier JB, Houlston R, Carvajal-Carmona L, Tomlinson I, Vine AE (2010). Genomic and geographic distribution of SNP-defined runs of homozygosity in Europeans. Hum Mol Genet.

