# Supplementary material for: Assessing runs of Homozygosity: a comparison of SNP Array and whole genome sequence low coverage data
Source: BMC Genomics. 2018 Jan 30;19:106. doi: 10.1186/s12864-018-4489-0 (PMC5789638; doi:10.1186/s12864-018-4489-0)
Supplement: Supplementary file 1 — Mean number of SNP (in homozygous state) per ROH in array data with 1 heterozygous SNP per ROH and WGS data with 1 to 5 heterozygous SNPs per ROH. ep(P,h) values for different populations P and allowed heterozygous SNP. (DOCX 22 kb) [file 12864_2018_4489_MOESM1_ESM.docx]

|  |  |  | Mean N SNP | *ep(P,h)* |  |
| --- | --- | --- | --- | --- | --- |
|  | FIN |  |  |  |  |
|  | Array. Het1 |  | 789.621 | 0.1266 |  |
|  | WGS. Het1 |  | 3196.544 | 0.0313 |  |
|  | WGS. Het2 |  | 3534.722 | 0.0566 |  |
|  | WGS. Het3 |  | 3504.332 | 0.0856 |  |
|  | WGS. Het4 |  | 3327.746 | 0.1202 |  |
|  | WGS. Het5 |  | 3192.579 | 0.1566 |  |
|  | GBR |  |  |  |  |
|  | Array. Het1 |  | 691.967 | 0.1445 |  |
|  | WGS. Het1 |  | 2808.204 | 0.0356 |  |
|  | WGS. Het2 |  | 3162.931 | 0.0632 |  |
|  | WGS. Het3 |  | 3108.546 | 0.0965 |  |
|  | WGS. Het4 |  | 2959.327 | 0.1352 |  |
|  | WGS. Het5 |  | 2865.225 | 0.1745 |  |
|  | IBS |  |  |  |  |
|  | Array. Het1 |  | 733.175 | 0.1364 |  |
|  | WGS. Het1 |  | 3088.600 | 0.0324 |  |
|  | WGS. Het2 |  | 3374.932 | 0.0593 |  |
|  | WGS. Het3 |  | 3350.463 | 0.0895 |  |
|  | WGS. Het4 |  | 3212.768 | 0.1245 |  |
|  | WGS. Het5 |  | 3040.110 | 0.1645 |  |
|  | TSI |  |  |  |  |
|  | Array. Het1 |  | 368.966 | 0.2710 |  |
|  | WGS. Het1 |  | 2830.188 | 0.0353 |  |
|  | WGS. Het2 |  | 3012.153 | 0.0664 |  |
|  | WGS. Het3 |  | 3006.729 | 0.0998 |  |
|  | WGS. Het4 |  | 2916.719 | 0.1371 |  |
|  | WGS. Het5 |  | 2788.239 | 0.1793 |  |
|  | CEU |  |  |  |  |
|  | Array. Het1 |  | 600.661 | 0.1665 |  |
|  | WGS. Het1 |  | 2898.197 | 0.0345 |  |
|  | WGS. Het2 |  | 2979.084 | 0.0671 |  |
|  | WGS. Het3 |  | 2951.206 | 0.1017 |  |
|  | WGS. Het4 |  | 2820.827 | 0.1418 |  |
|  | WGS. Het5 |  | 2740.967 | 0.1824 |  |
|  | ACB |  |  |  |  |
|  | Array. Het1 |  | 540.434 | 0.1850 |  |
|  | WGS. Het1 |  | 4148.352 | 0.0241 |  |
|  | WGS. Het2 |  | 4264.441 | 0.0469 |  |
|  | WGS. Het3 |  | 4333.085 | 0.0692 |  |
|  | WGS. Het4 |  | 4282.386 | 0.0934 |  |
|  | WGS. Het5 |  | 4101.131 | 0.1219 |  |
|  | ASW |  |  |  |  |
|  | Array. Het1 |  | 540.434 | 0.1850 |  |
|  | WGS. Het1 |  | 3588.354 | 0.0279 |  |
|  | WGS. Het2 |  | 4051.866 | 0.0494 |  |
|  | WGS. Het3 |  | 3959.986 | 0.0758 |  |
|  | WGS. Het4 |  | 3733.877 | 0.1071 |  |
|  | WGS. Het5 |  | 3619.036 | 0.1382 |  |
|  | MXL |  |  |  |  |
|  | Array. Het1 |  | 814.229 | 0.1228 |  |
|  | WGS. Het1 |  | 2770.899 | 0.0361 |  |
|  | WGS. Het2 |  | 3171.135 | 0.0631 |  |
|  | WGS. Het3 |  | 3283.340 | 0.0914 |  |
|  | WGS. Het4 |  | 3190.108 | 0.1254 |  |
|  | WGS. Het5 |  | 3101.256 | 0.1612 |  |
|  | CLM |  |  |  |  |
|  | Array. Het1 |  | 1205.452 | 0.0830 |  |
|  | WGS. Het1 |  | 3362.870 | 0.0297 |  |
|  | WGS. Het2 |  | 4397.076 | 0.0455 |  |
|  | WGS. Het3 |  | 4663.211 | 0.0643 |  |
|  | WGS. Het4 |  | 4535.970 | 0.0882 |  |
|  | WGS. Het5 |  | 4336.314 | 0.1153 |  |
|  | PEL |  |  |  |  |
|  | Array. Het1 |  | 862.721 | 0.1159 |  |
|  | WGS. Het1 |  | 2501.665 | 0.0400 |  |
|  | WGS. Het2 |  | 2772.508 | 0.0721 |  |
|  | WGS. Het3 |  | 2905.196 | 0.1033 |  |
|  | WGS. Het4 |  | 2881.633 | 0.1388 |  |
|  | WGS. Het5 |  | 2822.378 | 0.1772 |  |
|  | PUR |  |  |  |  |
|  | Array. Het1 |  | 973.355 | 0.1027 |  |
|  | WGS. Het1 |  | 3564.491 | 0.0281 |  |
|  | WGS. Het2 |  | 4197.875 | 0.0476 |  |
|  | WGS. Het3 |  | 4233.715 | 0.0709 |  |
|  | WGS. Het4 |  | 4096.342 | 0.0976 |  |
|  | WGS. Het5 |  | 3907.487 | 0.1280 |  |
|  | CDX |  |  |  |  |
|  | Array. Het1 |  | 785.479 | 0.1273 |  |
|  | WGS. Het1 |  | 3010.257 | 0.0332 |  |
|  | WGS. Het2 |  | 3320.151 | 0.0602 |  |
|  | WGS. Het3 |  | 3361.412 | 0.0892 |  |
|  | WGS. Het4 |  | 3192.388 | 0.1253 |  |
|  | WGS. Het5 |  | 3069.727 | 0.1629 |  |
|  | CHB |  |  |  |  |
|  | Array. Het1 |  | 594.195 | 0.1683 |  |
|  | WGS. Het1 |  | 2550.232 | 0.0392 |  |
|  | WGS. Het2 |  | 2487.692 | 0.0804 |  |
|  | WGS. Het3 |  | 2448.001 | 0.1225 |  |
|  | WGS. Het4 |  | 2390.554 | 0.1673 |  |
|  | WGS. Het5 |  | 2307.863 | 0.2167 |  |
|  | CHS |  |  |  |  |
|  | Array. Het1 |  | 607.864 | 0.1645 |  |
|  | WGS. Het1 |  | 2409.184 | 0.0415 |  |
|  | WGS. Het2 |  | 2432.931 | 0.0822 |  |
|  | WGS. Het3 |  | 2408.263 | 0.1246 |  |
|  | WGS. Het4 |  | 2340.319 | 0.1709 |  |
|  | WGS. Het5 |  | 2272.892 | 0.2200 |  |
|  | JPT |  |  |  |  |
|  | Array. Het1 |  | 624.679 | 0.1601 |  |
|  | WGS. Het1 |  | 2650.144 | 0.0377 |  |
|  | WGS. Het2 |  | 2824.324 | 0.0708 |  |
|  | WGS. Het3 |  | 2801.870 | 0.1071 |  |
|  | WGS. Het4 |  | 2729.735 | 0.1465 |  |
|  | WGS. Het5 |  | 2633.686 | 0.1898 |  |
|  | KHV |  |  |  |  |
|  | Array. Het1 |  | 639.120 | 0.1565 |  |
|  | WGS. Het1 |  | 2602.671 | 0.0384 |  |
|  | WGS. Het2 |  | 2665.047 | 0.0750 |  |
|  | WGS. Het3 |  | 2583.126 | 0.1161 |  |
|  | WGS. Het4 |  | 2438.045 | 0.1641 |  |
|  | WGS. Het5 |  | 2386.654 | 0.2095 |  |
|  | YRI |  |  |  |  |
|  | Array. Het1 |  | 722.610 | 0.1384 |  |
|  | WGS. Het1 |  | 4232.032 | 0.0236 |  |
|  | WGS. Het2 |  | 4677.840 | 0.0428 |  |
|  | WGS. Het3 |  | 4745.266 | 0.0632 |  |
|  | WGS. Het4 |  | 4585.748 | 0.0872 |  |
|  | WGS. Het5 |  | 4370.706 | 0.1144 |  |
|  | LWK |  |  |  |  |
|  | Array. Het1 |  | 839.470 | 0.1191 |  |
|  | WGS. Het1 |  | 4490.211 | 0.0223 |  |
|  | WGS. Het2 |  | 5216.283 | 0.0383 |  |
|  | WGS. Het3 |  | 5355.715 | 0.0560 |  |
|  | WGS. Het4 |  | 5224.002 | 0.0766 |  |
|  | WGS. Het5 |  | 4988.255 | 0.1002 |  |
|  | BAG |  |  |  |  |
|  | Array. Het1 |  | 622.591 | 0.1606 |  |
|  | WGS. Het1 |  | 3572.441 | 0.0280 |  |
|  | WGS. Het2 |  | 3935.833 | 0.0508 |  |
|  | WGS. Het3 |  | 3975.065 | 0.0755 |  |
|  | WGS. Het4 |  | 3874.708 | 0.1032 |  |
|  | WGS. Het5 |  | 3756.353 | 0.1331 |  |
|  | ZUL |  |  |  |  |
|  | Array. Het1 |  | 737.874 | 0.1355 |  |
|  | WGS. Het1 |  | 4064.322 | 0.0246 |  |
|  | WGS. Het2 |  | 4709.463 | 0.0425 |  |
|  | WGS. Het3 |  | 4995.259 | 0.0601 |  |
|  | WGS. Het4 |  | 4929.551 | 0.0811 |  |
|  | WGS. Het5 |  | 4770.573 | 0.1048 |  |
