# Supplementary material for: Assessing runs of Homozygosity: a comparison of SNP Array and whole genome sequence low coverage data
Source: BMC Genomics. 2018 Jan 30;19:106. doi: 10.1186/s12864-018-4489-0 (PMC5789638; doi:10.1186/s12864-018-4489-0)
Supplement: Supplementary file 2 — Pearson correlations (y-axis) of number of ROH, mean ROH size and mean sum of ROH between array data with 1 heterozygous allowed per RHO and WGS with 1 to 5 heterozygous SNP allowed per ROH (x-axis). (DOCX 28 kb) [file 12864_2018_4489_MOESM2_ESM.docx]

|  |  |  | Mean N of ROH | |  | Mean ROH Size | |  | Mean tot. sum ROH | |  |
| --- | --- | --- | --- | --- | --- | --- | --- | --- | --- | --- | --- |
|  |  |  | mean | Sd |  | mean | Sd |  | mean | Sd |  |
|  | FIN |  |  |  |  |  |  |  |  |  |  |
|  | Array. Het1 |  | 28.28 | 6.33 |  | 1 615.70 | 211.78 |  | 45 880.77 | 12.316.930 |  |
|  | WGS. Het1 |  | 7.19 | 3.72 |  | 1 365.59 | 307.16 |  | 9 727.88 | 5 280.02 |  |
|  | WGS. Het2 |  | 19.14 | 6.42 |  | 1 526.86 | 186.33 |  | 29 454.67 | 11 375.48 |  |
|  | WGS. Het3 |  | 28.10 | 6.62 |  | 1 562.15 | 195.36 |  | 44 185.65 | 13 018.38 |  |
|  | WGS. Het4 |  | 35.16 | 7.23 |  | 1 555.21 | 173.03 |  | 54 941.60 | 14 161.18 |  |
|  | WGS. Het5 |  | 40.92 | 7.67 |  | 1 543.81 | 154.79 |  | 63 400.65 | 14 659.85 |  |
|  | GBR |  |  |  |  |  |  |  |  |  |  |
|  | Array. Het1 |  | 23.49 | 5.00 |  | 1 499.09 | 408.37 |  | 35 414.50 | 13 067.90 |  |
|  | WGS. Het1 |  | 4.29 | 3.73 |  | 1 260.96 | 159.35 |  | 5 655.72 | 5 582.69 |  |
|  | WGS. Het2 |  | 14.25 | 6.43 |  | 1 399.94 | 242.30 |  | 21 017.33 | 14 689.40 |  |
|  | WGS. Het3 |  | 23.88 | 6.10 |  | 1 458.44 | 342.65 |  | 35 766.80 | 17 015.55 |  |
|  | WGS. Het4 |  | 30.69 | 6.39 |  | 1 459.10 | 338.02 |  | 45 504.95 | 17 700.96 |  |
|  | WGS. Het5 |  | 35.79 | 6.58 |  | 1 458.57 | 303.01 |  | 52 858.29 | 18 255.94 |  |
|  | IBS |  |  |  |  |  |  |  |  |  |  |
|  | Array. Het1 |  | 21.79 | 5.68 |  | 1 601.93 | 465.76 |  | 35 392.14 | 15 961.28 |  |
|  | WGS. Het1 |  | 4.66 | 4.51 |  | 1 307.09 | 221.34 |  | 6 431.56 | 6 809.21 |  |
|  | WGS. Het2 |  | 13.94 | 5.90 |  | 1 492.10 | 336.79 |  | 21 884.74 | 14 899.13 |  |
|  | WGS. Het3 |  | 22.03 | 6.11 |  | 1 561.10 | 436.66 |  | 35 133.26 | 17 791.16 |  |
|  | WGS. Het4 |  | 27.77 | 5.89 |  | 1 566.74 | 444.93 |  | 43 893.89 | 17 858.00 |  |
|  | WGS. Het5 |  | 32.78 | 6.01 |  | 1 538.83 | 380.26 |  | 50 892.28 | 18 357.23 |  |
|  | TSI |  |  |  |  |  |  |  |  |  |  |
|  | Array. Het1 |  | 18.67 | 4.26 |  | 1 492.49 | 381.00 |  | 27 991.84 | 983.26 |  |
|  | WGS. Het1 |  | 3.22 | 3.34 |  | 1 246.05 | 185.29 |  | 4 130.50 | 4 826.31 |  |
|  | WGS. Het2 |  | 10.87 | 5.19 |  | 1 377.72 | 260.15 |  | 15 811.04 | 13 280.97 |  |
|  | WGS. Het3 |  | 18.76 | 4.82 |  | 1 432.41 | 347.13 |  | 27 623.21 | 15 469.48 |  |
|  | WGS. Het4 |  | 24.83 | 5.03 |  | 1 445.07 | 349.53 |  | 36 510.46 | 16 249.73 |  |
|  | WGS. Het5 |  | 29.63 | 5.50 |  | 1 441.41 | 300.37 |  | 43 246.74 | 16 727.24 |  |
|  | CEU |  |  |  |  |  |  |  |  |  |  |
|  | Array. Het1 |  | 21.17 | 4.65 |  | 1 411.62 | 179.72 |  | 29 644.50 | 6 452.66 |  |
|  | WGS. Het1 |  | 3.12 | 3.89 |  | 1 276.45 | 199.03 |  | 4 021.19 | 5 553.51 |  |
|  | WGS. Het2 |  | 12.27 | 5.39 |  | 1 344.68 | 211.76 |  | 17 044.58 | 12 535.91 |  |
|  | WGS. Het3 |  | 21.16 | 5.30 |  | 1 401.33 | 318.55 |  | 30 125.84 | 14 479.10 |  |
|  | WGS. Het4 |  | 27.51 | 5.66 |  | 1 414.75 | 306.69 |  | 39 316.96 | 15 702.41 |  |
|  | WGS. Het5 |  | 32.60 | 5.87 |  | 1 423.45 | 320.32 |  | 46 631.80 | 15 836.02 |  |
|  | ACB |  |  |  |  |  |  |  |  |  |  |
|  | Array. Het1 |  | 8.11 | 3.05 |  | 1 542.43 | 394.69 |  | 12 674.15 | 7 110.44 |  |
|  | WGS. Het1 |  | 2.53 | 2.93 |  | 1 273.65 | 210.68 |  | 3 398.52 | 4 909.60 |  |
|  | WGS. Het2 |  | 5.27 | 3.42 |  | 1 371.88 | 281.93 |  | 7 703.91 | 8 018.26 |  |
|  | WGS. Het3 |  | 7.53 | 3.23 |  | 1 430.17 | 380.52 |  | 11 244.28 | 8 956.50 |  |
|  | WGS. Het4 |  | 8.99 | 3.28 |  | 1 487.75 | 630.10 |  | 13 619.64 | 9 015.97 |  |
|  | WGS. Het5 |  | 10.41 | 3.47 |  | 1 471.41 | 548.35 |  | 15 620.35 | 9 491.89 |  |
|  | ASW |  |  |  |  |  |  |  |  |  |  |
|  | Array. Het1 |  | 7.51 | 2.95 |  | 1 493.87 | 315.50 |  | 11 203.72 | 4 725.64 |  |
|  | WGS. Het1 |  | 1.83 | 1.80 |  | 1 190.68 | 137.28 |  | 2 292.82 | 2 509.06 |  |
|  | WGS. Het2 |  | 4.93 | 5.09 |  | 1 405.04 | 337.70 |  | 7 072.04 | 7 548.23 |  |
|  | WGS. Het3 |  | 7.15 | 6.81 |  | 1 416.01 | 273.95 |  | 10 202.68 | 9 870.73 |  |
|  | WGS. Het4 |  | 8.90 | 7.38 |  | 1 419.16 | 244.17 |  | 12 724.47 | 11 060.54 |  |
|  | WGS. Het5 |  | 10.56 | 8.13 |  | 1 418.34 | 238.97 |  | 15 032.42 | 11 963.35 |  |
|  | MXL |  |  |  |  |  |  |  |  |  |  |
|  | Array. Het1 |  | 27.43 | 8.54 |  | 1 657.49 | 703.89 |  | 47 061.72 | 32 685.51 |  |
|  | WGS. Het1 |  | 4.49 | 5.37 |  | 1 254.33 | 199.71 |  | 5 812.24 | 7 481.32 |  |
|  | WGS. Het2 |  | 17.95 | 13.00 |  | 1 408.80 | 262.84 |  | 26 945.80 | 26 841.72 |  |
|  | WGS. Het3 |  | 28.86 | 15.01 |  | 1 497.13 | 355.26 |  | 45 269.66 | 34 560.40 |  |
|  | WGS. Het4 |  | 36.69 | 15.89 |  | 1 520.18 | 401.88 |  | 57 536.29 | 36 907.60 |  |
|  | WGS. Het5 |  | 42.11 | 16.62 |  | 1 523.22 | 421.52 |  | 65 668.59 | 38 245.01 |  |
|  | CLM |  |  |  |  |  |  |  |  |  |  |
|  | Array. Het1 |  | 23.52 | 7.33 |  | 2 089.81 | 1 094.30 |  | 54 508.95 | 47 855.72 |  |
|  | WGS. Het1 |  | 9.01 | 11.84 |  | 1 336.09 | 197.87 |  | 12 898.28 | 18 192.83 |  |
|  | WGS. Het2 |  | 19.65 | 14.69 |  | 1 673.15 | 440.08 |  | 37 484.42 | 40 176.42 |  |
|  | WGS. Het3 |  | 25.83 | 10.56 |  | 1 848.67 | 686.90 |  | 53 021.73 | 45 012.63 |  |
|  | WGS. Het4 |  | 30.38 | 9.14 |  | 1 907.10 | 796.97 |  | 62 352.38 | 46 253.07 |  |
|  | WGS. Het5 |  | 34.45 | 8.95 |  | 1 890.08 | 796.19 |  | 69 027.26 | 46 918.18 |  |
|  | PEL |  |  |  |  |  |  |  |  |  |  |
|  | Array. Het1 |  | 52.98 | 14.62 |  | 1 612.74 | 751.07 |  | 86 978.69 | 56 542.13 |  |
|  | WGS. Het1 |  | 8.38 | 7.07 |  | 1 258.80 | 122.49 |  | 10 692.38 | 9 380.04 |  |
|  | WGS. Het2 |  | 31.52 | 18.18 |  | 1 384.17 | 170.63 |  | 45 430.18 | 36 777.32 |  |
|  | WGS. Het3 |  | 48.11 | 19.11 |  | 1 470.30 | 287.33 |  | 73 035.07 | 46 958.98 |  |
|  | WGS. Het4 |  | 59.16 | 20.88 |  | 1 507.62 | 406.22 |  | 90 956.13 | 50 396.78 |  |
|  | WGS. Het5 |  | 67.53 | 22.19 |  | 1 519.59 | 461.67 |  | 103 849.32 | 52 259.66 |  |
|  | PUR |  |  |  |  |  |  |  |  |  |  |
|  | Array. Het1 |  | 18.92 | 5.94 |  | 1 922.32 | 771.79 |  | 37 405.32 | 23 159.14 |  |
|  | WGS. Het1 |  | 5.53 | 6.07 |  | 1 373.24 | 235.43 |  | 7 803.70 | 9 180.86 |  |
|  | WGS. Het2 |  | 14.32 | 8.38 |  | 1 646.31 | 364.21 |  | 24 851.59 | 20 687.89 |  |
|  | WGS. Het3 |  | 20.27 | 6.50 |  | 1 757.78 | 522.49 |  | 37 019.20 | 22 829.46 |  |
|  | WGS. Het4 |  | 24.38 | 6.41 |  | 1 780.27 | 565.46 |  | 44 351.03 | 23 375.59 |  |
|  | WGS. Het5 |  | 27.85 | 6.73 |  | 1 763.93 | 580.41 |  | 49 824.36 | 23 741.34 |  |
|  | CDX |  |  |  |  |  |  |  |  |  |  |
|  | Array. Het1 |  | 31.84 | 5.74 |  | 1 637.84 | 418.26 |  | 52 728.33 | 19 173.67 |  |
|  | WGS. Het1 |  | 6.75 | 10.79 |  | 1 375.35 | 331.00 |  | 9 683.05 | 16 535.09 |  |
|  | WGS. Het2 |  | 18.26 | 12.89 |  | 1 537.89 | 362.60 |  | 30 779.51 | 35 390.78 |  |
|  | WGS. Het3 |  | 27.91 | 9.27 |  | 1 611.15 | 502.07 |  | 48 039.13 | 40 732.09 |  |
|  | WGS. Het4 |  | 35.44 | 7.54 |  | 1 623.18 | 613.55 |  | 59 903.76 | 41 271.55 |  |
|  | WGS. Het5 |  | 40.81 | 7.32 |  | 1 620.15 | 623.91 |  | 68 016.09 | 41 514.82 |  |
|  | CHB |  |  |  |  |  |  |  |  |  |  |
|  | Array. Het1 |  | 26.81 | 4.97 |  | 1 364.54 | 162.26 |  | 36 731.86 | 8 863.08 |  |
|  | WGS. Het1 |  | 3.02 | 3.13 |  | 1 234.45 | 158.46 |  | 3 835.76 | 4 612.79 |  |
|  | WGS. Het2 |  | 13.45 | 5.30 |  | 1 299.83 | 205.38 |  | 18 063.69 | 12 064.46 |  |
|  | WGS. Het3 |  | 23.48 | 5.29 |  | 1 349.61 | 245.65 |  | 32 251.56 | 13 968.67 |  |
|  | WGS. Het4 |  | 31.33 | 5.31 |  | 1 382.89 | 251.47 |  | 43 664.60 | 14 380.31 |  |
|  | WGS. Het5 |  | 37.24 | 5.35 |  | 1 387.18 | 226.77 |  | 51 957.31 | 14 514.52 |  |
|  | CHS |  |  |  |  |  |  |  |  |  |  |
|  | Array. Het1 |  | 28.13 | 4.37 |  | 1 385.64 | 187.26 |  | 38 996.22 | 8 196.10 |  |
|  | WGS. Het1 |  | 3.02 | 2.26 |  | 1 206.87 | 160.53 |  | 3 716.87 | 3 081.71 |  |
|  | WGS. Het2 |  | 13.48 | 4.29 |  | 1 303.63 | 181.28 |  | 17 807.76 | 7 801.68 |  |
|  | WGS. Het3 |  | 24.49 | 4.61 |  | 1 348.61 | 185.85 |  | 33 268.15 | 9 858.94 |  |
|  | WGS. Het4 |  | 32.60 | 5.19 |  | 1 362.21 | 164.71 |  | 44 596.89 | 10 633.45 |  |
|  | WGS. Het5 |  | 38.61 | 5.71 |  | 1 372.61 | 146.97 |  | 53 142.16 | 11 149.22 |  |
|  | JPT |  |  |  |  |  |  |  |  |  |  |
|  | Array. Het1 |  | 29.75 | 5.80 |  | 1 414.21 | 310.31 |  | 42 320.23 | 14 913.61 |  |
|  | WGS. Het1 |  | 4.82 | 7.71 |  | 1 262.21 | 168.35 |  | 6 395.09 | 11 861.48 |  |
|  | WGS. Het2 |  | 15.03 | 12.80 |  | 1 340.30 | 222.37 |  | 22 264.02 | 30 263.29 |  |
|  | WGS. Het3 |  | 25.97 | 8.55 |  | 1 410.82 | 412.41 |  | 39 346.31 | 35 617.47 |  |
|  | WGS. Het4 |  | 34.53 | 7.21 |  | 1 455.66 | 520.03 |  | 52 351.99 | 36 466.65 |  |
|  | WGS. Het5 |  | 41.24 | 6.91 |  | 1 467.31 | 551.43 |  | 62 068.26 | 36 417.81 |  |
|  | KHV |  |  |  |  |  |  |  |  |  |  |
|  | Array. Het1 |  | 28.92 | 4.41 |  | 1 416.21 | 224.97 |  | 41 122.13 | 11 506.36 |  |
|  | WGS. Het1 |  | 3.17 | 3.26 |  | 1 270.81 | 201.49 |  | 4 053.56 | 4 640.69 |  |
|  | WGS. Het2 |  | 12.60 | 4.97 |  | 1 340.27 | 194.64 |  | 17 173.05 | 9 077.81 |  |
|  | WGS. Het3 |  | 23.61 | 5.51 |  | 1 369.13 | 192.90 |  | 32 584.26 | 10 674.44 |  |
|  | WGS. Het4 |  | 32.22 | 6.09 |  | 1 377.75 | 184.72 |  | 44 571.67 | 11 733.94 |  |
|  | WGS. Het5 |  | 38.69 | 6.01 |  | 1 386.65 | 173.84 |  | 53 725.77 | 11 582.47 |  |
|  | YRI |  |  |  |  |  |  |  |  |  |  |
|  | Array. Het1 |  | 10.74 | 4.04 |  | 1 579.32 | 434.24 |  | 17 247.42 | 9 127.31 |  |
|  | WGS. Het1 |  | 2.79 | 2.46 |  | 1 278.35 | 172.48 |  | 3 698.29 | 3 596.71 |  |
|  | WGS. Het2 |  | 6.49 | 3.39 |  | 1 454.22 | 355.93 |  | 9 823.41 | 6 897.47 |  |
|  | WGS. Het3 |  | 9.08 | 4.03 |  | 1 519.90 | 410.17 |  | 14 009.77 | 8 054.87 |  |
|  | WGS. Het4 |  | 10.89 | 4.25 |  | 1 518.15 | 393.84 |  | 16 729.19 | 8 646.20 |  |
|  | WGS. Het5 |  | 12.48 | 4.37 |  | 1 522.56 | 355.99 |  | 19 167.56 | 8 966.82 |  |
|  | LWK |  |  |  |  |  |  |  |  |  |  |
|  | Array. Het1 |  | 11.76 | 3.39 |  | 1 702.41 | 304.36 |  | 20 017.00 | 6 613.31 |  |
|  | WGS. Het1 |  | 3.56 | 2.06 |  | 1 375.64 | 280.19 |  | 4 886.67 | 3 003.97 |  |
|  | WGS. Het2 |  | 8.40 | 3.19 |  | 1 580.00 | 298.02 |  | 13 321.43 | 6 069.59 |  |
|  | WGS. Het3 |  | 10.77 | 3.39 |  | 1 670.01 | 379.42 |  | 17 954.88 | 7 028.74 |  |
|  | WGS. Het4 |  | 12.23 | 3.51 |  | 1 659.32 | 333.03 |  | 20 357.29 | 7 500.39 |  |
|  | WGS. Het5 |  | 13.75 | 3.57 |  | 1 632.77 | 289.42 |  | 22 553.36 | 7 628.85 |  |
|  | BAG |  |  |  |  |  |  |  |  |  |  |
|  | Array. Het1 |  | 12.58 | 4.21 |  | 1 647.82 | 258.30 |  | 21 009.13 | 8 416.62 |  |
|  | WGS. Het1 |  | 5.54 | 3.05 |  | 1 345.31 | 167.97 |  | 7 584.75 | 4 442.98 |  |
|  | WGS. Het2 |  | 10.12 | 4.06 |  | 1 493.53 | 199.58 |  | 15 543.23 | 7 402.98 |  |
|  | WGS. Het3 |  | 13.92 | 4.61 |  | 1 560.71 | 177.81 |  | 22 069.64 | 8 678.70 |  |
|  | WGS. Het4 |  | 16.63 | 4.69 |  | 1 601.28 | 171.64 |  | 26 860.93 | 8 852.83 |  |
|  | WGS. Het5 |  | 18.88 | 4.85 |  | 1 625.07 | 181.15 |  | 30 824.30 | 9 216.79 |  |
|  | ZUL |  |  |  |  |  |  |  |  |  |  |
|  | Array. Het1 |  | 16.15 | 4.25 |  | 1 885.40 | 304.84 |  | 30 452.25 | 9 665.20 |  |
|  | WGS. Het1 |  | 6.71 | 3.28 |  | 1 349.52 | 164.09 |  | 9 152.49 | 4 709.10 |  |
|  | WGS. Het2 |  | 13.42 | 4.61 |  | 1 572.06 | 224.73 |  | 21 242.43 | 8 090.21 |  |
|  | WGS. Het3 |  | 16.94 | 4.80 |  | 1 712.61 | 239.04 |  | 29 116.77 | 9 372.11 |  |
|  | WGS. Het4 |  | 19.52 | 4.70 |  | 1 756.42 | 236.40 |  | 34 369.27 | 9 726.83 |  |
|  | WGS. Het5 |  | 21.22 | 4.81 |  | 1 780.14 | 222.54 |  | 37 865.96 | 10 082.98 |  |
