# Supplementary material for: Assessing runs of Homozygosity: a comparison of SNP Array and whole genome sequence low coverage data
Source: BMC Genomics. 2018 Jan 30;19:106. doi: 10.1186/s12864-018-4489-0 (PMC5789638; doi:10.1186/s12864-018-4489-0)
Supplement: Supplementary file 3 — Means and standard deviations of number of ROH, ROH size and total sum of ROH for different populations, technologies and allowed heterozygous SNPs per ROH (DOCX 35 kb) [file 12864_2018_4489_MOESM3_ESM.docx]

|  |  |  | Mean N of ROH | | | |  | Mean ROH Size | | | |  | Mean tot. sum ROH | | | |  |
| --- | --- | --- | --- | --- | --- | --- | --- | --- | --- | --- | --- | --- | --- | --- | --- | --- | --- |
|  |  |  | Correlation | |  | MWW |  | Correlation | |  | MWW |  | Correlation | |  | MWW |  |
|  |  |  | Pear. | P-val |  | P-val |  | Pear. | P-val |  | P-val |  | Pear. | P-val |  | P-val |  |
|  | FIN |  |  |  |  |  |  |  |  |  |  |  |  |  |  |  |  |
|  | WGS. Het1 |  | 0.4143 | 0.0000 |  | 0.0000 |  | 0.2810 | 0.0067 |  | 0.0000 |  | 0.5843 | 0.0000 |  | 0.0000 |  |
|  | WGS. Het2 |  | 0.6625 | 0.0000 |  | 0.0000 |  | 0.7081 | 0.0000 |  | 0.0003 |  | 0.8289 | 0.0000 |  | 0.0000 |  |
|  | WGS. Het3 |  | 0.8310 | 0.0000 |  | 0.0777 |  | 0.9094 | 0.0000 |  | 0.0858 |  | 0.9148 | 0.0000 |  | 0.8522 |  |
|  | WGS. Het4 |  | 0.8289 | 0.0000 |  | 0.0000 |  | 0.9181 | 0.0000 |  | 0.0100 |  | 0.9145 | 0.0000 |  | 0.0000 |  |
|  | WGS. Het5 |  | 0.8218 | 0.0000 |  | 0.0000 |  | 0.9156 | 0.0000 |  | 0.0026 |  | 0.9025 | 0.0000 |  | 0.0000 |  |
|  | GBR |  |  |  |  |  |  |  |  |  |  |  |  |  |  |  |  |
|  | WGS. Het1 |  | 0.3064 | 0.0064 |  | 0.0000 |  | 0.3750 | 0.0007 |  | 0.0000 |  | 0.8036 | 0.0000 |  | 0.0000 |  |
|  | WGS. Het2 |  | 0.5610 | 0.0000 |  | 0.0000 |  | 0.8912 | 0.0000 |  | 0.0017 |  | 0.8788 | 0.0000 |  | 0.0000 |  |
|  | WGS. Het3 |  | 0.7091 | 0.0000 |  | 0.0785 |  | 0.9671 | 0.0000 |  | 0.0767 |  | 0.9173 | 0.0000 |  | 0.2651 |  |
|  | WGS. Het4 |  | 0.7373 | 0.0000 |  | 0.0000 |  | 0.9693 | 0.0000 |  | 0.1465 |  | 0.9203 | 0.0000 |  | 0.0000 |  |
|  | WGS. Het5 |  | 0.7279 | 0.0000 |  | 0.0000 |  | 0.9742 | 0.0000 |  | 0.2824 |  | 0.9133 | 0.0000 |  | 0.0000 |  |
|  | IBS |  |  |  |  |  |  |  |  |  |  |  |  |  |  |  |  |
|  | WGS. Het1 |  | 0.3485 | 0.0007 |  | 0.0000 |  | 0.4754 | 0.0000 |  | 0.0000 |  | 0.7894 | 0.0000 |  | 0.0000 |  |
|  | WGS. Het2 |  | 0.6368 | 0.0000 |  | 0.0000 |  | 0.8199 | 0.0000 |  | 0.0065 |  | 0.9334 | 0.0000 |  | 0.0000 |  |
|  | WGS. Het3 |  | 0.7908 | 0.0000 |  | 0.0587 |  | 0.9380 | 0.0000 |  | 0.1272 |  | 0.9510 | 0.0000 |  | 0.3167 |  |
|  | WGS. Het4 |  | 0.8059 | 0.0000 |  | 0.0000 |  | 0.9685 | 0.0000 |  | 0.2840 |  | 0.9526 | 0.0000 |  | 0.0000 |  |
|  | WGS. Het5 |  | 0.8200 | 0.0000 |  | 0.0000 |  | 0.9643 | 0.0000 |  | 0.2247 |  | 0.9535 | 0.0000 |  | 0.0000 |  |
|  | TSI |  |  |  |  |  |  |  |  |  |  |  |  |  |  |  |  |
|  | WGS. Het1 |  | 0.1211 | 0.2975 |  | 0.0000 |  | 0.2575 | 0.0247 |  | 0.0000 |  | 0.5683 | 0.0000 |  | 0.0000 |  |
|  | WGS. Het2 |  | 0.5251 | 0.0000 |  | 0.0000 |  | 0.7676 | 0.0000 |  | 0.0009 |  | 0.8207 | 0.0000 |  | 0.0000 |  |
|  | WGS. Het3 |  | 0.6770 | 0.0000 |  | 0.0637 |  | 0.8923 | 0.0000 |  | 0.0855 |  | 0.8984 | 0.0000 |  | 0.8040 |  |
|  | WGS. Het4 |  | 0.6941 | 0.0000 |  | 0.0000 |  | 0.9230 | 0.0000 |  | 0.5660 |  | 0.8987 | 0.0000 |  | 0.0000 |  |
|  | WGS. Het5 |  | 0.6694 | 0.0000 |  | 0.0000 |  | 0.9335 | 0.0000 |  | 0.8832 |  | 0.8883 | 0.0000 |  | 0.0000 |  |
|  | CEU |  |  |  |  |  |  |  |  |  |  |  |  |  |  |  |  |
|  | WGS. Het1 |  | 0.2750 | 0.0119 |  | 0.0000 |  | 0.1838 | 0.0962 |  | 0.0000 |  | 0.3644 | 0.0007 |  | 0.0000 |  |
|  | WGS. Het2 |  | 0.5763 | 0.0000 |  | 0.0000 |  | 0.4754 | 0.0000 |  | 0.0000 |  | 0.6432 | 0.0000 |  | 0.0000 |  |
|  | WGS. Het3 |  | 0.7043 | 0.0000 |  | 0.0987 |  | 0.7600 | 0.0000 |  | 0.0578 |  | 0.7320 | 0.0000 |  | 0.2571 |  |
|  | WGS. Het4 |  | 0.7138 | 0.0000 |  | 0.0000 |  | 0.7299 | 0.0000 |  | 0.2549 |  | 0.7666 | 0.0000 |  | 0.0000 |  |
|  | WGS. Het5 |  | 0.7324 | 0.0000 |  | 0.0000 |  | 0.7878 | 0.0000 |  | 0.1082 |  | 0.7702 | 0.0000 |  | 0.0000 |  |
|  | ACB |  |  |  |  |  |  |  |  |  |  |  |  |  |  |  |  |
|  | WGS. Het1 |  | 0.4076 | 0.0013 |  | 0.0000 |  | 0.1715 | 0.0857 |  | 0.0000 |  | 0.8607 | 0.0008 |  | 0.0000 |  |
|  | WGS. Het2 |  | 0.7310 | 0.0000 |  | 0.0000 |  | 0.7136 | 0.0002 |  | 0.0000 |  | 0.9218 | 0.0000 |  | 0.0000 |  |
|  | WGS. Het3 |  | 0.8157 | 0.0000 |  | 0.9807 |  | 0.8683 | 0.0000 |  | 0.0588 |  | 0.9422 | 0.0000 |  | 0.3259 |  |
|  | WGS. Het4 |  | 0.7970 | 0.0000 |  | 0.0046 |  | 0.8955 | 0.0000 |  | 0.0309 |  | 0.9161 | 0.0000 |  | 0.0500 |  |
|  | WGS. Het5 |  | 0.7563 | 0.0000 |  | 0.0000 |  | 0.9315 | 0.0000 |  | 0.0173 |  | 0.9031 | 0.0000 |  | 0.0000 |  |
|  | ASW |  |  |  |  |  |  |  |  |  |  |  |  |  |  |  |  |
|  | WGS. Het1 |  | 0.1220 | 0.5791 |  | 0.0000 |  | 0.2455 | 0.2589 |  | 0.0000 |  | 0.3065 | 0.1549 |  | 0.0000 |  |
|  | WGS. Het2 |  | 0.4872 | 0.0030 |  | 0.0000 |  | 0.5577 | 0.0005 |  | 0.0288 |  | 0.6296 | 0.0001 |  | 0.0000 |  |
|  | WGS. Het3 |  | 0.5973 | 0.0001 |  | 0.5336 |  | 0.8760 | 0.0000 |  | 0.0853 |  | 0.6836 | 0.0000 |  | 0.2689 |  |
|  | WGS. Het4 |  | 0.6179 | 0.0000 |  | 0.0277 |  | 0.8540 | 0.0000 |  | 0.1343 |  | 0.7119 | 0.0000 |  | 0.1799 |  |
|  | WGS. Het5 |  | 0.6733 | 0.0000 |  | 0.0000 |  | 0.8736 | 0.0000 |  | 0.1406 |  | 0.7597 | 0.0000 |  | 0.0004 |  |
|  | MXL |  |  |  |  |  |  |  |  |  |  |  |  |  |  |  |  |
|  | WGS. Het1 |  | 0.2800 | 0.1145 |  | 0.0000 |  | 0.1388 | 0.4412 |  | 0.0000 |  | 0.9154 | 0.0000 |  | 0.0000 |  |
|  | WGS. Het2 |  | 0.5044 | 0.0011 |  | 0.0000 |  | 0.8111 | 0.0000 |  | 0.0005 |  | 0.9572 | 0.0000 |  | 0.0000 |  |
|  | WGS. Het3 |  | 0.7169 | 0.0000 |  | 0.8181 |  | 0.9359 | 0.0000 |  | 0.0918 |  | 0.9798 | 0.0000 |  | 0.5526 |  |
|  | WGS. Het4 |  | 0.8360 | 0.0000 |  | 0.0000 |  | 0.9864 | 0.0000 |  | 0.3472 |  | 0.9843 | 0.0000 |  | 0.0034 |  |
|  | WGS. Het5 |  | 0.8633 | 0.0000 |  | 0.0000 |  | 0.9945 | 0.0000 |  | 0.3534 |  | 0.9841 | 0.0000 |  | 0.0000 |  |
|  | CLM |  |  |  |  |  |  |  |  |  |  |  |  |  |  |  |  |
|  | WGS. Het1 |  | 0.6629 | 0.0000 |  | 0.0000 |  | 0.3867 | 0.0035 |  | 0.0000 |  | 0.9387 | 0.0000 |  | 0.0000 |  |
|  | WGS. Het2 |  | 0.7447 | 0.0000 |  | 0.0001 |  | 0.8533 | 0.0000 |  | 0.0081 |  | 0.9879 | 0.0000 |  | 0.0001 |  |
|  | WGS. Het3 |  | 0.8550 | 0.0000 |  | 0.0965 |  | 0.9730 | 0.0000 |  | 0.1955 |  | 0.9942 | 0.0000 |  | 0.8955 |  |
|  | WGS. Het4 |  | 0.9036 | 0.0000 |  | 0.0000 |  | 0.9898 | 0.0000 |  | 0.2767 |  | 0.9947 | 0.0000 |  | 0.0062 |  |
|  | WGS. Het5 |  | 0.8842 | 0.0000 |  | 0.0000 |  | 0.9905 | 0.0000 |  | 0.2630 |  | 0.9938 | 0.0000 |  | 0.0000 |  |
|  | PEL |  |  |  |  |  |  |  |  |  |  |  |  |  |  |  |  |
|  | WGS. Het1 |  | 0.2963 | 0.0508 |  | 0.0000 |  | 0.2110 | 0.1691 |  | 0.0000 |  | 0.8543 | 0.0000 |  | 0.0000 |  |
|  | WGS. Het2 |  | 0.4661 | 0.0013 |  | 0.0000 |  | 0.7499 | 0.0000 |  | 0.0000 |  | 0.9687 | 0.0000 |  | 0.0000 |  |
|  | WGS. Het3 |  | 0.7923 | 0.0000 |  | 0.1652 |  | 0.9650 | 0.0000 |  | 0.0875 |  | 0.9880 | 0.0000 |  | 0.0987 |  |
|  | WGS. Het4 |  | 0.9421 | 0.0000 |  | 0.0405 |  | 0.9948 | 0.0000 |  | 0.0806 |  | 0.9943 | 0.0000 |  | 0.1856 |  |
|  | WGS. Het5 |  | 0.9634 | 0.0000 |  | 0.0000 |  | 0.9977 | 0.0000 |  | 0.2028 |  | 0.9962 | 0.0000 |  | 0.0006 |  |
|  | PUR |  |  |  |  |  |  |  |  |  |  |  |  |  |  |  |  |
|  | WGS. Het1 |  | 0.2858 | 0.0282 |  | 0.0000 |  | 0.1219 | 0.3575 |  | 0.0000 |  | 0.8445 | 0.0000 |  | 0.0000 |  |
|  | WGS. Het2 |  | 0.5293 | 0.0000 |  | 0.0000 |  | 0.6199 | 0.0000 |  | 0.0007 |  | 0.9470 | 0.0000 |  | 0.0000 |  |
|  | WGS. Het3 |  | 0.7679 | 0.0000 |  | 0.0579 |  | 0.9291 | 0.0000 |  | 0.0542 |  | 0.9744 | 0.0000 |  | 0.6079 |  |
|  | WGS. Het4 |  | 0.8589 | 0.0000 |  | 0.0000 |  | 0.9741 | 0.0000 |  | 0.0566 |  | 0.9820 | 0.0000 |  | 0.0004 |  |
|  | WGS. Het5 |  | 0.8681 | 0.0000 |  | 0.0000 |  | 0.9856 | 0.0000 |  | 0.0333 |  | 0.9822 | 0.0000 |  | 0.0000 |  |
|  | CDX |  |  |  |  |  |  |  |  |  |  |  |  |  |  |  |  |
|  | WGS. Het1 |  | 0.2620 | 0.0205 |  | 0.0000 |  | 0.4288 | 0.0001 |  | 0.0000 |  | 0.8400 | 0.0000 |  | 0.0000 |  |
|  | WGS. Het2 |  | 0.5529 | 0.0000 |  | 0.0000 |  | 0.8311 | 0.0000 |  | 0.0047 |  | 0.9213 | 0.0000 |  | 0.0000 |  |
|  | WGS. Het3 |  | 0.6903 | 0.0000 |  | 0.0018 |  | 0.9387 | 0.0000 |  | 0.1082 |  | 0.9493 | 0.0000 |  | 0.0875 |  |
|  | WGS. Het4 |  | 0.7549 | 0.0000 |  | 0.0000 |  | 0.9688 | 0.0000 |  | 0.1211 |  | 0.9593 | 0.0000 |  | 0.0053 |  |
|  | WGS. Het5 |  | 0.8147 | 0.0000 |  | 0.0000 |  | 0.9697 | 0.0000 |  | 0.2015 |  | 0.9706 | 0.0000 |  | 0.0000 |  |
|  | CHB |  |  |  |  |  |  |  |  |  |  |  |  |  |  |  |  |
|  | WGS. Het1 |  | 0.1674 | 0.1213 |  | 0.0000 |  | 0.3247 | 0.0022 |  | 0.0000 |  | 0.5348 | 0.0000 |  | 0.0000 |  |
|  | WGS. Het2 |  | 0.5653 | 0.0000 |  | 0.0000 |  | 0.6631 | 0.0000 |  | 0.0000 |  | 0.7556 | 0.0000 |  | 0.0000 |  |
|  | WGS. Het3 |  | 0.6784 | 0.0000 |  | 0.0473 |  | 0.8360 | 0.0000 |  | 0.0579 |  | 0.7975 | 0.0000 |  | 0.0988 |  |
|  | WGS. Het4 |  | 0.7372 | 0.0000 |  | 0.0000 |  | 0.9092 | 0.0000 |  | 0.5338 |  | 0.8272 | 0.0000 |  | 0.0000 |  |
|  | WGS. Het5 |  | 0.7459 | 0.0000 |  | 0.0000 |  | 0.9202 | 0.0000 |  | 0.6898 |  | 0.8392 | 0.0000 |  | 0.0000 |  |
|  | CHS |  |  |  |  |  |  |  |  |  |  |  |  |  |  |  |  |
|  | WGS. Het1 |  | 0.2360 | 0.0545 |  | 0.0000 |  | 0.3146 | 0.0095 |  | 0.0000 |  | 0.5956 | 0.0000 |  | 0.0000 |  |
|  | WGS. Het2 |  | 0.4446 | 0.0000 |  | 0.0000 |  | 0.8549 | 0.0000 |  | 0.0000 |  | 0.7006 | 0.0000 |  | 0.0000 |  |
|  | WGS. Het3 |  | 0.5844 | 0.0000 |  | 0.0216 |  | 0.9044 | 0.0000 |  | 0.0782 |  | 0.8171 | 0.0000 |  | 0.0013 |  |
|  | WGS. Het4 |  | 0.6484 | 0.0000 |  | 0.0000 |  | 0.9294 | 0.0000 |  | 0.0905 |  | 0.8264 | 0.0000 |  | 0.0000 |  |
|  | WGS. Het5 |  | 0.6665 | 0.0000 |  | 0.0000 |  | 0.9211 | 0.0000 |  | 0.5201 |  | 0.8372 | 0.0000 |  | 0.0000 |  |
|  | JPT |  |  |  |  |  |  |  |  |  |  |  |  |  |  |  |  |
|  | WGS. Het1 |  | 0.3526 | 0.0014 |  | 0.0000 |  | 0.0424 | 0.7106 |  | 0.0000 |  | 0.7747 | 0.0000 |  | 0.0000 |  |
|  | WGS. Het2 |  | 0.3397 | 0.0008 |  | 0.0000 |  | 0.6845 | 0.0000 |  | 0.0000 |  | 0.8297 | 0.0000 |  | 0.0000 |  |
|  | WGS. Het3 |  | 0.5610 | 0.0000 |  | 0.0010 |  | 0.9367 | 0.0000 |  | 0.0528 |  | 0.8740 | 0.0000 |  | 0.0589 |  |
|  | WGS. Het4 |  | 0.6838 | 0.0000 |  | 0.0000 |  | 0.9697 | 0.0000 |  | 0.0563 |  | 0.9080 | 0.0000 |  | 0.0000 |  |
|  | WGS. Het5 |  | 0.7211 | 0.0000 |  | 0.0000 |  | 0.9813 | 0.0000 |  | 0.2781 |  | 0.9171 | 0.0000 |  | 0.0000 |  |
|  | KHV |  |  |  |  |  |  |  |  |  |  |  |  |  |  |  |  |
|  | WGS. Het1 |  | 0.2798 | 0.0120 |  | 0.0000 |  | 0.1660 | 0.1410 |  | 0.0000 |  | 0.6950 | 0.0000 |  | 0.0000 |  |
|  | WGS. Het2 |  | 0.5905 | 0.0000 |  | 0.0000 |  | 0.7571 | 0.0000 |  | 0.0000 |  | 0.8558 | 0.0000 |  | 0.0000 |  |
|  | WGS. Het3 |  | 0.7325 | 0.0000 |  | 0.0000 |  | 0.9004 | 0.0000 |  | 0.0589 |  | 0.8999 | 0.0000 |  | 0.0528 |  |
|  | WGS. Het4 |  | 0.7771 | 0.0000 |  | 0.0000 |  | 0.9365 | 0.0000 |  | 0.0291 |  | 0.9044 | 0.0000 |  | 0.0000 |  |
|  | WGS. Het5 |  | 0.7753 | 0.0000 |  | 0.0000 |  | 0.9479 | 0.0000 |  | 0.2217 |  | 0.9112 | 0.0000 |  | 0.0000 |  |
|  | YRI |  |  |  |  |  |  |  |  |  |  |  |  |  |  |  |  |
|  | WGS. Het1 |  | 0.4287 | 0.0001 |  | 0.0000 |  | 0.3370 | 0.0027 |  | 0.0000 |  | 0.7684 | 0.0000 |  | 0.0000 |  |
|  | WGS. Het2 |  | 0.7448 | 0.0000 |  | 0.0000 |  | 0.7814 | 0.0000 |  | 0.0003 |  | 0.9109 | 0.0000 |  | 0.0000 |  |
|  | WGS. Het3 |  | 0.8686 | 0.0000 |  | 0.0559 |  | 0.8919 | 0.0000 |  | 0.0589 |  | 0.9517 | 0.0000 |  | 0.0590 |  |
|  | WGS. Het4 |  | 0.8965 | 0.0000 |  | 0.1248 |  | 0.9525 | 0.0000 |  | 0.0666 |  | 0.9628 | 0.0000 |  | 0.4826 |  |
|  | WGS. Het5 |  | 0.8886 | 0.0000 |  | 0.0000 |  | 0.9538 | 0.0000 |  | 0.1690 |  | 0.9573 | 0.0000 |  | 0.0025 |  |
|  | LWK |  |  |  |  |  |  |  |  |  |  |  |  |  |  |  |  |
|  | WGS. Het1 |  | 0.3744 | 0.0025 |  | 0.0000 |  | 0.0849 | 0.5084 |  | 0.0000 |  | 0.5782 | 0.0000 |  | 0.0000 |  |
|  | WGS. Het2 |  | 0.7461 | 0.0000 |  | 0.0000 |  | 0.6923 | 0.0000 |  | 0.0002 |  | 0.8608 | 0.0000 |  | 0.0000 |  |
|  | WGS. Het3 |  | 0.7703 | 0.0000 |  | 0.6460 |  | 0.8188 | 0.0000 |  | 0.0650 |  | 0.9142 | 0.0000 |  | 0.1805 |  |
|  | WGS. Het4 |  | 0.8030 | 0.0000 |  | 0.0221 |  | 0.9028 | 0.0000 |  | 0.0811 |  | 0.9041 | 0.0000 |  | 0.3047 |  |
|  | WGS. Het5 |  | 0.8327 | 0.0000 |  | 0.0000 |  | 0.8810 | 0.0000 |  | 0.0354 |  | 0.9185 | 0.0000 |  | 0.0023 |  |
|  | BAG |  |  |  |  |  |  |  |  |  |  |  |  |  |  |  |  |
|  | WGS. Het1 |  | 0.2596 | 0.2316 |  | 0.0000 |  | 0.3659 | 0.0859 |  | 0.0000 |  | 0.4922 | 0.0170 |  | 0.0000 |  |
|  | WGS. Het2 |  | 0.3173 | 0.0000 |  | 0.0097 |  | 0.7116 | 0.0000 |  | 0.0000 |  | 0.6180 | 0.0000 |  | 0.0003 |  |
|  | WGS. Het3 |  | 0.5263 | 0.1222 |  | 0.0088 |  | 0.6850 | 0.0001 |  | 0.0516 |  | 0.7065 | 0.0010 |  | 0.0920 |  |
|  | WGS. Het4 |  | 0.7028 | 0.0057 |  | 0.0000 |  | 0.7793 | 0.0001 |  | 0.2365 |  | 0.8203 | 0.0001 |  | 0.0000 |  |
|  | WGS. Het5 |  | 0.7741 | 0.0001 |  | 0.0000 |  | 0.8365 | 0.0000 |  | 0.5968 |  | 0.8414 | 0.0000 |  | 0.0000 |  |
|  | ZUL |  |  |  |  |  |  |  |  |  |  |  |  |  |  |  |  |
|  | WGS. Het1 |  | 0.6186 | 0.0000 |  | 0.0000 |  | 0.3468 | 0.0038 |  | 0.0000 |  | 0.7586 | 0.0000 |  | 0.0000 |  |
|  | WGS. Het2 |  | 0.8465 | 0.0000 |  | 0.0003 |  | 0.8765 | 0.0000 |  | 0.0000 |  | 0.9236 | 0.0000 |  | 0.0000 |  |
|  | WGS. Het3 |  | 0.8361 | 0.0000 |  | 0.0518 |  | 0.6888 | 0.0000 |  | 0.0452 |  | 0.9068 | 0.0000 |  | 0.6823 |  |
|  | WGS. Het4 |  | 0.8540 | 0.0000 |  | 0.0000 |  | 0.8249 | 0.0000 |  | 0.0004 |  | 0.9261 | 0.0000 |  | 0.0008 |  |
|  | WGS. Het5 |  | 0.8960 | 0.0000 |  | 0.0000 |  | 0.8954 | 0.0000 |  | 0.0058 |  | 0.9423 | 0.0000 |  | 0.0000 |  |
