# Supplementary figures and images for: Assessing runs of Homozygosity: a comparison of SNP Array and whole genome sequence low coverage data
Source: BMC Genomics. 2018 Jan 30;19:106. doi: 10.1186/s12864-018-4489-0 (PMC5789638; doi:10.1186/s12864-018-4489-0)

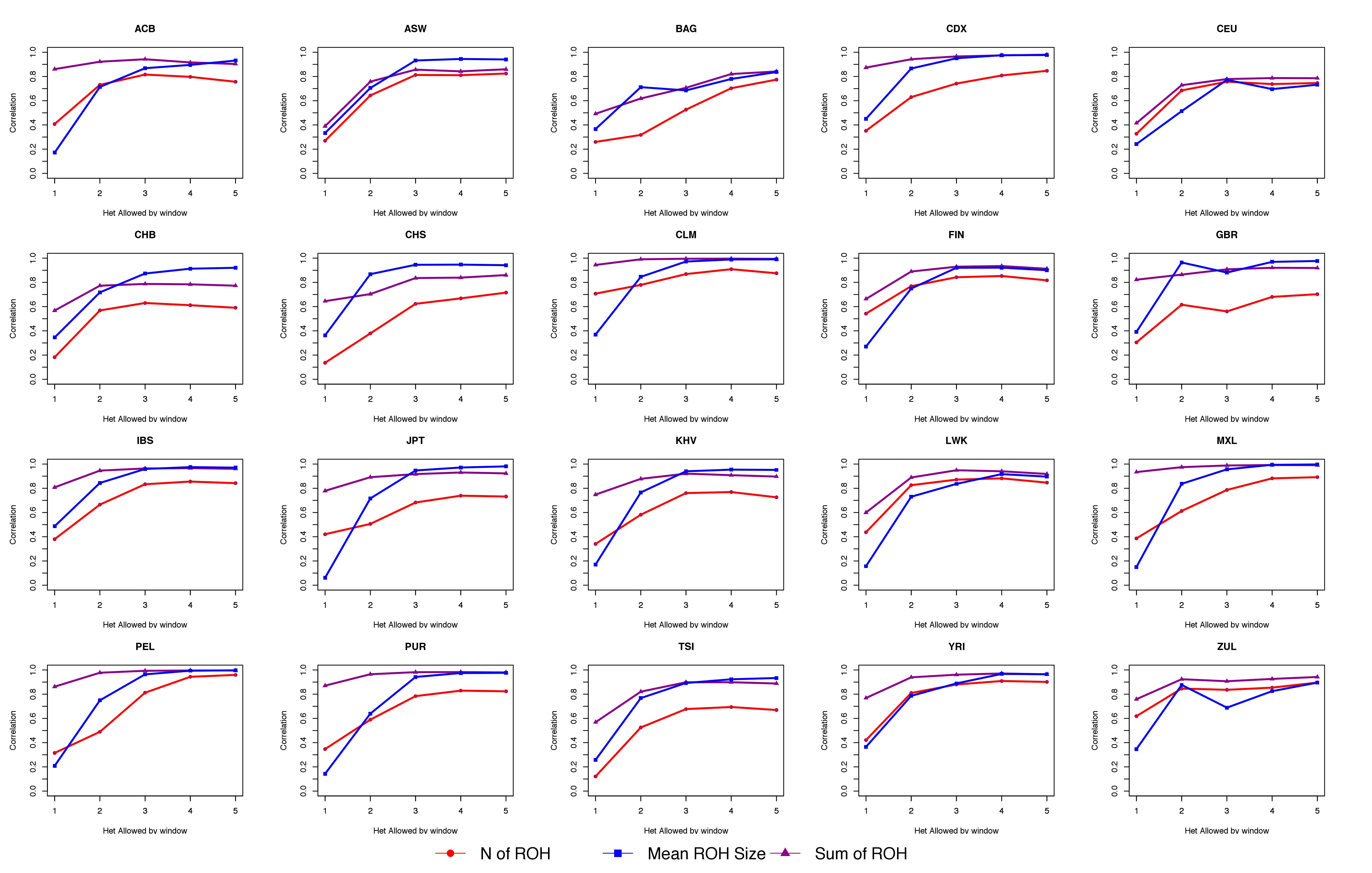

Supplement: Supplementary file 4 — Pearson correlations (with p-values) and Mann-Whitney-Wilcoxon non- parametrical test p vales between array data with 1 heterozygous SNP per ROH and WGS with 1 to 5 heterozygous SNPs per ROH. (JPEG 1011 kb) [file 12864_2018_4489_MOESM4_ESM.jpeg]
